# Metasurface analogues of molecular diastereomers from hierarchical multiscale chiral interactions with biomolecules

**DOI:** 10.1038/s41467-026-72200-6

**Published:** 2026-04-22

**Authors:** Dominic J. P. Koyroytsaltis-McQuire, Shailendra K. Chaubey, Rahul Kumar, Paula L. Lalaguna, Tamas Javorfi, Giuliano Siligardi, Affar Karimullah, Adrian J. Lapthorn, Yoshito Y. Tanaka, Shun Hashiyada, Nikolaj Gadegaard, Malcolm Kadodwala

**Affiliations:** 1 School of Chemistry, University of Glasgow, Glasgow, UK; 2 Diamond Light Source Ltd., Harwell Science and Innovation Campus, Didcot, UK; 3 Research Institute for Electronic Science, Hokkaido University, Sapporo, Hokkaido Japan; 4 PRESTO, Japan Science and Technology Agency, Kawaguchi, Saitama, Japan; 5 School of Engineering, Rankine Building, University of Glasgow, Glasgow, UK

**Keywords:** Circular dichroism, Metamaterials

## Abstract

Chirality occurs at molecular and nanoscales, but these forms of handedness are usually treated independently. We demonstrate a hybrid chiral state, termed a meta-diastereomer, in which molecular chirality and nanoscale structural chirality are coupled at an interface to determine the optical response, with distinct states defined by the relative handedness of the two components, as in molecular diastereomers. Unlike strategies that rely on enhanced near-field optical chirality, the coupling here arises from a charged, polarisable interfacial layer that perturbs electromagnetic boundary conditions. The resulting reduction in symmetry renders linear optical observables stereostructurally informative, in the same way that molecular diastereomers can be distinguished using non-chiral measurement techniques. This behaviour distinguishes native versus denatured protein layers and differentiates protein-protein binding states in a model antigen-antibody system. These results identify electromagnetic boundary conditions as a mechanism by which chirality can be transmitted across length scales and provide a boundary-condition-based framework for hierarchical chirality at biomolecule-metasurface interfaces.

## Abstract (web summary)

Many of life’s molecules are chiral and they exist in mirror-image forms. Here, the authors show that proteins attached to chiral nanostructures create hybrid states capable of distinguishing between different protein forms without the need for chemical labels.

## Introduction

Chirality, the property of an object being non-superimposable on its mirror image, is a fundamental organising principle across chemistry^1^, biology^2^, and physics^3^. In biomolecules, chirality is inherently hierarchical^4,5^: local stereocentres define secondary structure, which organises tertiary and quaternary architecture and ultimately governs biological function. At larger length scales, chirality can also be encoded into inorganic nanostructures, enabling control over light polarisation, optical spin, and chiroptical response^6–11^. Despite extensive advances at both molecular and nanoscale levels, chirality is most often treated as a scale-specific property^12–15^.

A key unresolved question is how molecular-scale chirality can influence and be transduced into the optical response of nanoscale photonic systems. Addressing this issue is essential for understanding hierarchical chirality, where chiral order emerges through coupling across distinct length scales rather than within a single structural level^4^.

Here we show that adsorption of charged chiral biomolecules onto enantiomorphic silicon metasurfaces generates symmetry-inequivalent hybrid states through interfacial perturbation of electromagnetic boundary conditions, thereby transmitting molecular chirality into the far-field optical response.

This mechanism can be understood as a boundary-value effect^16–20^. Adsorption creates a charged, polarisable chiral interfacial layer comprising the molecule, its hydration shell, and associated counter-ions, which modifies the electromagnetic boundary conditions experienced by the resonator modes. Chirality is therefore transmitted across length scales at the interface itself, rather than through engineered near-field enhancement^21–27^ or bulk chiral constitutive descriptions^28–31^.

**Fig. 1:**
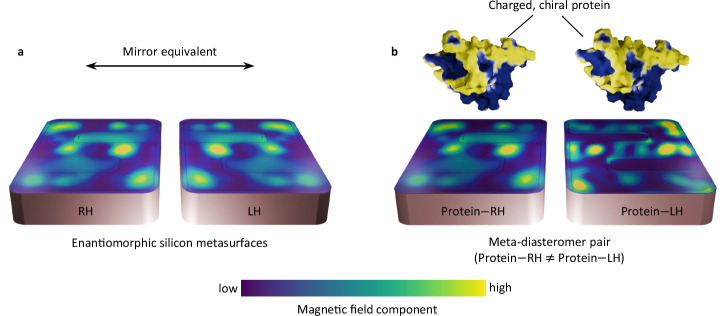
Concept of meta-diastereomer formation. **a** LH and RH silicon metasurfaces are mirror-equivalent in the absence of molecular adsorption. **b** Binding of a charged, chiral protein layer modifies the electromagnetic boundary conditions at the interface, breaking mirror equivalence and forming a pair of symmetry-inequivalent hybrid states (Protein-LH ≠ Protein-RH), termed meta-diastereomers. The colour scale of the protein represents the surface charge, and the image size is not to scale.

We term these hybrid states ‘meta-diastereomers’ (Fig. 1). In direct analogy to molecular diastereomers, binding to left-handed (LH) and right-handed (RH) nanostructures generates symmetry-inequivalent states (Protein-LH ≠ Protein-RH) whose optical response reflects the combined handedness of molecule and resonator. In this symmetry-reduced system, linear dichroism becomes stereostructurally informative.

Unlike superchiral enhancement strategies that amplify weak circular dichroism signals^25,32–34^ meta-diastereomer formation does not rely on enhanced optical chirality. Instead, chirality is transmitted through interfacial boundary perturbation, rendering linear observables sensitive to molecular stereostructure.

Using enantiomorphic silicon metasurfaces coupled to model protein systems, we demonstrate meta-diastereomer formation and isolate their optical signatures through polarisation-resolved measurements. By combining experiment with phenomenological modelling, we show that conventional constitutive descriptions fail to capture the observed behaviour, whereas a boundary-condition framework naturally accounts for the emergence of stereostructural sensitivity.

## Results and Discussion

We first establish the mirror-equivalent baseline optical response of the enantiomorphic silicon metasurfaces before examining the effects of protein adsorption.

### Refractive-index control and protein binding (reflectance)

**Fig. 2:**
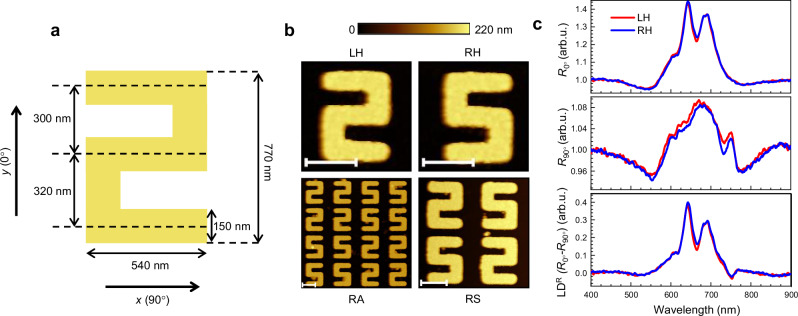
Mirror-equivalent baseline optical response of enantiomorphic metasurfaces. **a** Schematic of the planar S-shaped silicon resonator defining the long (0°) and short (90°) optical axes. The periodicity of the metasurface is 850 nm. **b** AFM images of LH, RH, racemic-array (RA), and racemic-site (RS) metasurfaces, confirming structural enantiomorphism and dimensional equivalence (scale bars: 400 nm). **c** Polarisation-dependent reflectance spectra measured in buffer at 0° and 90° linear polarisations together with the corresponding reflectance-derived linear dichroism, LD^R^ = *R*_0°_ − *R*_90°_. The reflectance and LD^R^ responses are indistinguishable for LH (red) and RH (blue) metasurfaces in buffer, confirming mirror-equivalent baseline behaviour arising solely from structural anisotropy. Source data are provided as a Source Data file.

The metasurfaces consist of asymmetric double split-ring resonators with an S-shaped geometry (Fig. 2a), fabricated from achiral amorphous silicon. The S geometry defines S_LH_ and S_RH_ enantiomorphic forms of the resonator, while the unequal spacing between the upper, middle and lower arms removes rotational symmetry and places the nanostructure geometry in the C₁ point group. In addition to arrays composed exclusively of S_LH_ or S_RH_ resonators, two racemic configurations were fabricated. In the racemic-site (RS) design, S_LH_ and S_RH_ structures alternate site-by-site, whereas in the racemic array (RA) design the metasurface comprises 19 × 19 single-handed subdomains. AFM imaging confirmed structural enantiomorphism and dimensional equivalence across metasurfaces types (Fig. 2b). While we optimise the fabrication process for a thickness of 180 nm, actual thickness ranges from 170 to 210 nm across different runs. Although these unavoidable sample-to-sample variations produce spectral shifts and line-shape differences, they do not affect the qualitative behaviour described below.

The optical response of the metasurfaces was characterised using reflectance spectroscopy and Müller Matrix Polarimetry (MMP) measured in transmission. Reflectance spectra were acquired using linearly polarised light aligned either along the long axis (0°) or the short axis (90°) (Fig. 2a) of the resonator. The difference between the 0° and 90° reflectance spectra is denoted as the reflectance-derived linear dichroism, (LD^R^). The raw differential MMP data were decomposed into circular dichroism (CD), circular birefringence (CB), linear dichroism along 0° and 90° axes (LD’) and along ± 45° (LD”), linear birefringence LB’ and LB”, dissymmetry factor (*g*-factor = 2(Abs_L_-Abs_R_)/(Abs_L_+Abs_R_)), absorbance (Abs) and depolarization index (0 ≤ DI ≤ 1) using the analytic inversion method^35^. Films of DI = 1 are non-depolarising chiral materials whilst DI = 0 are depolarising. The analytic inversion method is valid with 0.5 ≤ DI ≤ 1 but not for DI lower than 0.5 that indicates the complexity of the materials investigated from which the chiroptical and optical properties cannot be fully decomposed (Supplementary Figs. 5-13).

As reported previously, the 0° reflectance spectra (Fig. 2c) of the LH and RH structures exhibit a pronounced doublet centred at ≈655 nm^11^, arising predominantly from the magnetic-dipole resonance of the S-structures^11^. The corresponding 90° spectra (Fig. 2c) also display this resonance but with reduced intensity and a modified line shape, reflecting the different current pathways excited along the short axis of the resonator. For each polarisation state (0° and 90°), the reflectance spectra of LH and RH metasurfaces are essentially identical, resulting in indistinguishable LD^R^ spectra; only minor deviations attributable to fabrication imperfections are observed (Fig. 2c). This confirms that reflectance-derived linear dichroism is the same for both handednesses, as expected for a quantity that is not intrinsically chirally sensitive.

A complete MMP study of the bare substrates has been reported in ref. ^11^. It was shown that the enantiomorphic metasurfaces exhibit equal and opposite CD, while their LD′ responses are identical^11^. In contrast, the ±45° channel (LD″) changes sign when the handedness is switched and when the propagation direction is reversed. This behaviour is incompatible with reciprocity and therefore indicates that LD″ originates from anisotropic birefringence rather than optical activity^36–38^. Further height-dependent multipole analysis confirmed that the dominant resonances arise from magnetic-dipole and higher-order multipolar modes.

**Fig. 3:**
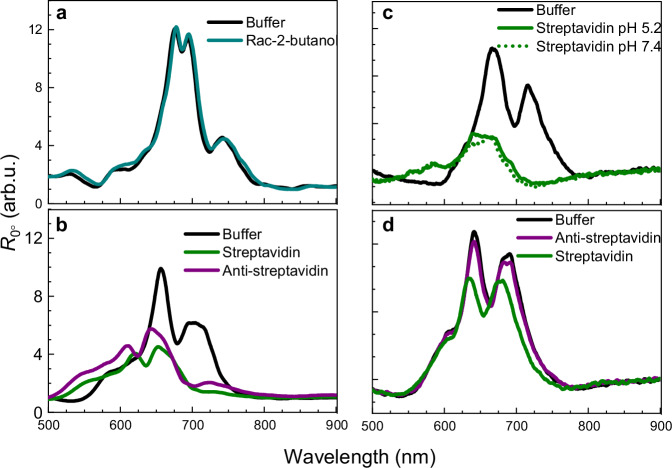
Reflectance response of S_LH_ metasurfaces under dielectric and biomolecular modification. Reflectance spectra of representative S_LH_ silicon metasurfaces, S_RH_ data in Supplementary Fig. 17, with the silicon thickness of each sample given in parentheses. Spectra collected with the metasurfaces immersed PBS buffer are shown in black. These are compared with: **a** (210 nm) immersion in rac-2-butanol (dark cyan); **b** (190 nm) immobilised streptavidin (green) followed by binding of anti-streptavidin (purple); **c** (180 nm) immobilised denatured streptavidin measured at pH 5.2 (green) and after exchange back to pH 7.4 buffer (dotted green); and (**d**) (170 nm) immobilised anti-streptavidin (purple) followed by subsequent binding of streptavidin (green). All spectra were acquired in buffer after each functionalisation or binding step. Source data are provided as a Source Data file.

To benchmark the refractive-index sensitivity of the metasurfaces, we compared their 0° reflectance spectra in two dielectric environments: phosphate-buffered saline (PBS, pH 7.4, refractive index 1.339) and racemic 2-butanol (refractive index 1.397) (Fig. 3a). Immersion in rac-2-butanol produces a small red shift of ≈3 nm in the magnetic-dipole resonance, consistent with the modest increase in the surrounding refractive index. This corresponds to a sensitivity of ≈48 nm RIU⁻¹, more than an order of magnitude lower than values typically reported for plasmonic resonances, reflecting the intrinsically weak refractive-index dependence of high-index dielectric resonators.

Reflectance-derived spectra acquired in rac-2-butanol, (R)−2-butanol and (S)−2-butanol exhibit the same small red shift and remain indistinguishable for LH and RH metasurfaces, with no measurable asymmetries within experimental reproducibility (Supplementary Fig. 1). These neutral-molecule controls therefore establish a baseline in which dielectric loading produces only a small red shift and no handedness-dependent linear dichroism for non-charged chiral molecules.

Protein-metasurface hybrids were formed using a lysine-based covalent coupling scheme to immobilise streptavidin (strept) and anti-streptavidin (anti-strept) on the S-structures^39^. Poly-L-lysine (PLL) was first adsorbed onto the silicon surface to provide a positively charged layer, followed by glutaraldehyde cross-linking of surface amines to lysine residues on the protein. Residual aldehyde groups were capped with ethanolamine to minimise non-specific adsorption (see Methods). Strept-functionalised metasurfaces were subsequently incubated with polyclonal anti-strept to form a strept-anti-strept complex, while anti-strept-functionalised metasurfaces were incubated with strept to generate the reciprocal assembly. Protein immobilisation did not change the morphology of the metasurfaces, as confirmed by AFM (Supplementary Fig. 4). Denatured strept layers were prepared by immobilising strept as above and then incubating the metasurfaces in pH 5.2 buffer, providing a control in which the interfacial protein layer is preserved but its native higher-order structure is disrupted.

Protein binding breaks the mirror equivalence of the S_LH_ and S_RH_, and accordingly handedness-dependent differences are present in the underlying reflectance spectra. We do not emphasise these differences in the raw reflectance plots because they are most reliably and transparently visualised in the LD^R^, which is computed directly from the same 0° and 90° reflectance measurements and suppresses common-mode spectral contributions. For clarity, we therefore present reflectance spectra for a single silicon enantiomorph, S_LH_, to illustrate the overall spectral shifts and gross intensity changes associated with protein binding, while the handedness-dependent anisotropic response is quantified using the MMP linear dichroism measurements reported in the subsequent sections.

Immobilisation of strept produces a clear blue shift of ≈15 nm in the dominant magnetic-dipole resonance in the 0° reflectance spectra (Fig. 3b), accompanied by a redistribution of intensity within the doublet. This behaviour was replicated in substrates with silicon thicknesses of 180 and 210 nm. This behaviour is opposite in sign to the red shift expected from increased local refractive index. Denaturation of the strept layer at pH 5.2 leads to a pronounced change in reflectance line shape, with the magnetic-dipole doublet collapsing into a narrower feature with slight splitting, but without any additional blue shift of the trailing edge of the resonance (Fig. 3c). The denaturation of strept is irreversible^40^, consequently the only significant effect of changing the buffer pH from 5.2 to 7.4, given the pI ≈ 6, is to change the biomolecules surface charge from net positive to net negative. This causes both slight increase in intensity and modification of the line shape of the remnant magnetic dipole resonance.

Anti-strept immobilisation also induces a blue shift of the magnetic-dipole resonance, but with a smaller magnitude of ≈1 nm and only subtle changes in the relative intensities of the doublet (Fig. 3d).

Subsequent binding steps produce further blue shifts of ≈2 nm when anti-strept binds to an immobilised strept (Fig. 3d), and ≈7 nm when strept binds to an immobilised anti-strept (Fig. 3d) Overall, the binding response mirrors that observed upon immobilising the proteins individually, with strept consistently producing larger shifts than anti-strept.

The reflectance data impose two constraints on the origin of the blue shift. First, the magnitude of the blue shift increases systematically with the build-up of the interfacial protein layer. Second, the blue shift is insensitive to the sign of the protein’s net charge. Strept has an isoelectric point near pH 5–6 and therefore changes its net charge sign across this range, yet the blue shift persists under conditions where the net charge is reversed. This behaviour is inconsistent with a simple surface-dipole layer whose polarity is set by the sign of the fixed charge. Although changes in pH can also influence adsorption and hydration, the opposite sign relative to neutral refractive-index loading and the systematic scaling with layer thickness constrain the origin of the effect to an interfacial perturbation rather than bulk dielectric loading.

Both observations are consistent with models in which the charged protein layer, its hydration shell, and associated counter-ions form a highly polarisable interfacial region that modifies the electromagnetic boundary conditions at the silicon surface^16,19,20,41,42^. Treatments of charged interfacial layers in achiral systems predict resonance blue shifts which is insensitive to the net charge of the layer^17^. Within this framework, the interfacial region acts as a thin, highly polarisable boundary that reduces the effective dielectric contrast at the silicon interface, giving rise to the observed blue shift. The larger shifts measured for sequential strept and anti-strept binding therefore reflect an increase in the thickness and polarisability of the interfacial region rather than changes in protein charge or bulk refractive index.

### Müller-matrix polarimetry: LD and optical activity

Transmission MMP provides a direct experimental test for meta-diastereomer formation by separating birefringent and optically active contributions. For mirror-equivalent enantiomorphic metasurfaces, symmetry requires identical linear dichroism; therefore, the emergence of handedness-dependent LD following protein binding constitutes the defining experimental signature of meta-diastereomer formation.

**Fig. 4:**
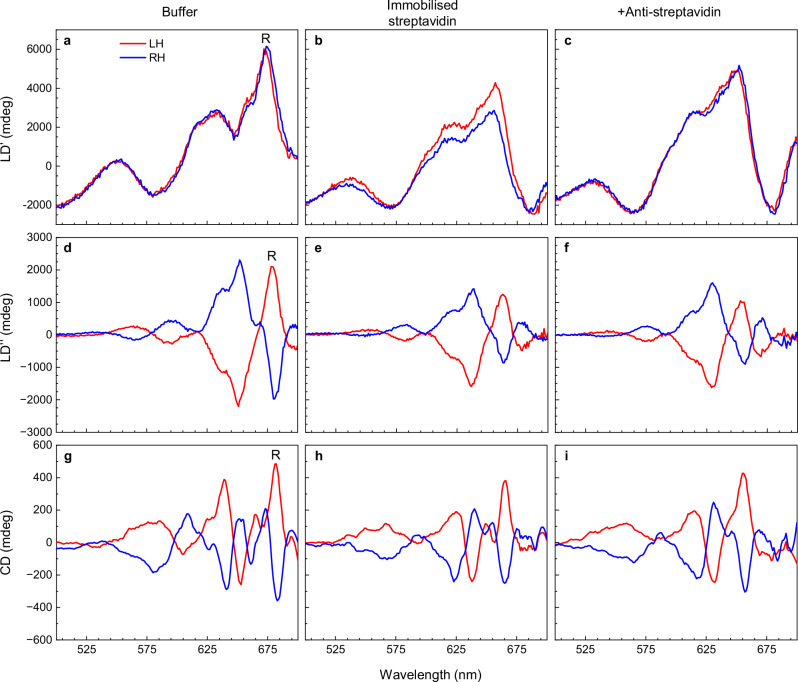
Müller-matrix polarimetry reveals handedness-dependent optical response after protein binding. Müller-matrix-derived spectra for S_LH_ (red lines) and S_RH_ (blue lines) metasurfaces. **a–c** Linear dichroism LD’ (0°/90° channel) in (**a**) buffer, (**b**) immobilised streptavidin and (**c**) after anti-streptavidin binding. **d–f** Linear dichroism LD” (± 45° channel) in (**d**) buffer, (**e**) immobilised streptavidin and (**f**) after anti-streptavidin binding. **g–i** Circular dichroism CD in (**g**) buffer, (**h**) immobilised streptavidin and (**i**) after anti-streptavidin binding. The dominant magnetic-dipole resonance used for quantitative analysis is indicated by R. Source data are provided as a Source Data file.

We therefore acquired MMP data for the metasurfaces after immobilisation of strept and again following binding of anti-strept to the strept layer. The resulting LD′, LD″, and CD spectra for LH and RH structures are shown in Fig. 4. To quantify the divergence between S_LH_ and S_RH_, we define an asymmetry parameter $\alpha,$ Eq. (1), where ${R}_{{{\rm{LH}}}}$ and ${R}_{{{\rm{RH}}}}$ are the values for LH and RH metasurfaces, respectively; equal to the ratio of the LD′, LD″, and CD values at the resonance associated with the dominant magnetic-dipole mode (peak R in Fig. 4).

1 $${{\rm{\alpha }}}=\frac{{R}_{{{\rm{LH}}}}}{{R}_{{{\rm{RH}}}}},$$

The extracted values are reported in Table 1.

**Table 1:** Asymmetry parameters

	Asymmetry parameter	Values
CD	${\alpha }_{{{\rm{Buffer}}}}^{{{\rm{CD}}}}$	1.37 ± 0.04
	${\alpha }_{{{\rm{Strept}}}}^{{{\rm{CD}}}}$	1.56 ± 0.04
LD’	${\alpha }_{{{\rm{Buffer}}}}^{{{{\rm{LD}}}}^{{\prime} }}$	0.99 ± 0.02
	${\alpha }_{{{\rm{Strept}}}}^{{{{\rm{LD}}}}^{{\prime} }}$	1.51 ± 0.02
LD”	${\alpha }_{{{\rm{Buffer}}}}^{{{{\rm{LD}}}}^{{\prime} {\prime} }}$	1.07 ± 0.02
	${\alpha }_{{{\rm{Strept}}}}^{{{{\rm{LD}}}}^{{\prime} {\prime} }}$	1.44 ± 0.02

Asymmetry parameters extracted at resonance R of Fig. 4., quantifying the divergence between LH and RH responses and demonstrating loss of mirror equivalence following streptavidin binding. The α parameter is defined in Eq. (1) and errors are derived from 4 repeated measurements.

Strept produces the largest perturbation, introducing clear differences in the magnitudes of the magnetic-dipole resonance in LD′, LD″, and CD for LH and RH metasurfaces. The most pronounced effect is observed in LD′, where the two enantiomers, which exhibit identical LD′ responses in buffer, develop markedly different amplitudes following strept binding.

CD also displays handedness-dependent changes upon protein binding. However, its absolute magnitude in the buffer state deviates from unity due to depolarisation and residual linear anisotropy intrinsic to the low-symmetry S-structures. In MMP, LD′, and LD″ are extracted from the Müller matrix as linear-dichroic terms, whereas CD is extracted as the circular-dichroic term (see Methods), allowing these contributions to be assessed separately within the same measurement. In depolarising systems, Müller-matrix-derived CD can contain an achiral offset arising from multiple scattering and polarisation mixing, even in the absence of intrinsic optical activity. In such cases, the Müller-matrix-derived CD obtained by the Analytic Inversion method can acquire an achiral offset when the depolarisation Index (DI) is low, reflecting multiple scattering and polarisation mixing rather than intrinsic optical activity. In our analysis we therefore use the MMP decomposition to treat LD′ and LD″ explicitly as the linear-dichroic terms and CD as the circular-dichroic term, and we interpret CD primarily through its changes upon binding rather than its absolute value. Consistent with this, the differential Müller-matrix elements show opposite sign for *M*_03_ and *M*_30_ (non-reciprocal circular response) (Supplementary Figs. 5–13), while elements expected to be anti-symmetric can exhibit symmetry in depolarising systems. By contrast, the transition from identical LD responses in buffer to divergent LD responses after protein binding directly reflects loss of mirror equivalence and is not subject to the same ambiguity. For this reason, we focus on changes in CD upon protein binding rather than its absolute value. By contrast, linear dichroism is not subject to this ambiguity: the transition from identical LD responses in buffer to divergent LD responses after protein binding directly reflects the loss of mirror equivalence between the two metasurface enantiomers.

The racemic RA and RS metasurfaces (Supplementary Fig. 2) exhibit dichroic responses intermediate between those of the two enantiomorphs and follow the same systematic trends upon strept and anti-strept binding. This confirms that the observed effects are intrinsic to the protein-nanostructure interaction rather than artefacts of array ordering. Non-zero CD in the racemic controls is therefore not unexpected.

Having separated birefringent and optically active contributions, we now focus on LD^R^ as a practical stereostructural readout and examine how its resonance features diverge between LH and RH metasurfaces upon binding.

### Reflectance linear dichroism signatures of meta-diastereomers

MMP provides the most rigorous separation of birefringent and optically active contributions. However, full Müller-matrix acquisition and analysis are experimentally demanding. To complement these measurements and to demonstrate a simpler, scalable readout, we therefore examine LD^R^, which directly captures changes in the linear optical response of the metasurfaces upon protein adsorption.

We use LD^R^ to track the optical response associated with immobilised native strept, denatured strept, anti-strept, and the subsequent binding of strept to an immobilised anti-strept layer. These binding events induce clear asymmetries in the LD^R^ response. Although the strept–anti-strept interaction is not of intrinsic pathological interest, it provides a well-defined and controllable model antigen–antibody system. As such, it captures the essential features of a diagnostic binding assay and serves to illustrate the broader applicability of the meta-diastereomer concept.

**Fig. 5:**
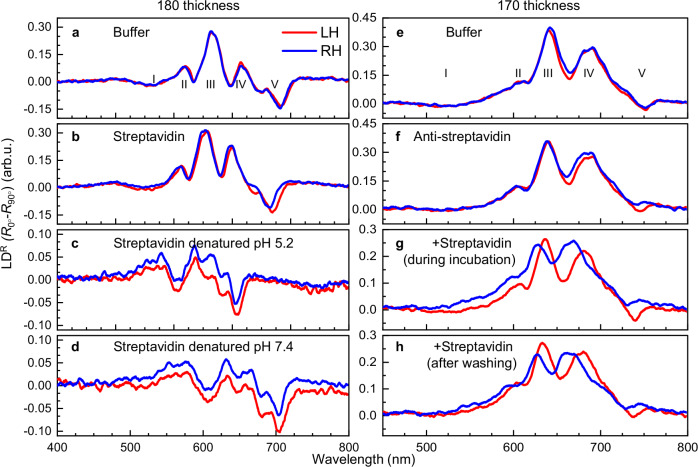
Reflectance-derived linear dichroism under different protein-binding conditions. Reflectance-derived LD^R^ for enantiomorphic metasurfaces, showing LH (red) and RH (blue) responses. **a**–**d** Metasurface with nominal silicon thickness 180 nm, measured sequentially in (**a**) buffer, (**b**) after streptavidin immobilisation (**c**) after streptavidin denaturation (pH 5.2), and (**d)** after buffer (pH 7.4) exchange following denaturation. **e-h** Metasurface with nominal silicon thickness 170 nm, measured sequentially in (**e**) buffer, (**f**) after anti-streptavidin immobilisation, (**g**) in the presence of streptavidin solution (binding to the immobilised anti-streptavidin layer), and (**h**) after washing and re-measuring in buffer (pH 7.4). Five resonances have been labelled as I-V. Source data are provided as a Source Data file.

Reflectance-derived LD^R^ spectra for immobilised native and denatured strept (Fig. 5a), as well as for anti-strept (Fig. 5b), were acquired on metasurface arrays fabricated with slightly different silicon thicknesses. While the overall spectral structure is qualitatively similar across batches, systematic differences are evident. Each substrate exhibits three positive resonances between 600 and 720 nm (labelled II–IV) and two negative resonances centred at 500–550 nm (I) and 750–780 nm (V), with resonance I being much weaker than resonance V. These features are present in all samples but differ in linewidth and relative intensity, with narrower resonances observed for the substrate used in the strept experiments.

Immobilisation of strept (Fig. 5a) produces a clear blue shift of the LD^R^ resonances, consistent with the reflectance spectra, and introduces a pronounced asymmetry between S_LH_ and S_RH_ metasurfaces. For all resonances, systematically larger blue shifts are observed for S_RH_ compared to S_LH_. In addition, resonances I and V display distinct handedness-dependent differences in relative intensity. Changing the buffer pH from 7.4 to 5.2, which denatures the immobilised strept, leads to a substantial modification of the LD^R^ line shape. Under these conditions, resonances I–IV remain approximately aligned, whereas resonance V is strongly quenched.

Immobilisation of anti-strept (Fig. 5b) produces a smaller blue shift than strept and introduces asymmetries in both resonance position and intensity. Subsequent binding of strept to anti-strept-functionalised metasurfaces has a significantly larger effect on the LD^R^ spectra than the initial anti-strept immobilisation. This binding step results in an enhanced blue shift and pronounced handedness-dependent differences in both resonance positions and relative intensities. Measurements acquired with the metasurfaces immersed in the strept solution, and after replacement with buffer, show that strept binding consistently shifts the S_LH_ resonances to longer wavelengths than the corresponding S_RH_ resonances. Following buffer exchange, the overall asymmetry decreases slightly, consistent with partial dissociation of bound strept. As for immobilised anti-strept and the anti-strept/strept complexes, the blue shifts are again larger for S_RH_ than for S_LH_ metasurfaces.

### Theory of the boundary-condition model

To formalise the physical mechanism by which molecular and nanostructure chirality are electromagnetically coupled to form meta-diastereomers, we rationalise meta-diastereomer formation using an interfacial electrodynamics description in which protein adsorption creates a thin, structured, chiral, polarisable boundary layer. Through modified electromagnetic boundary conditions, molecular stereostructure is encoded into an effective two-dimensional chiral interfacial response that perturbs the resonant modes of the enantiomorphic silicon metasurfaces. In this view, the meta-diastereomer is formed at the interface: molecular stereostructure modifies the optical response through coupled modifications of the normal and tangential field-matching conditions that define the silicon metasurface resonances. We first outline the boundary-condition model and show how an interfacial description accounts for both the observed blue shift of the resonances and the formation of meta-diastereomers upon molecular binding. We then use numerical simulations to test whether this minimal interfacial perturbation is sufficient to reproduce the key experimental signatures.

Pasteur Model: Effects of chiral dielectrics on nanophotonic systems have, up to this point, most commonly been described using the Pasteur constitutive formalism^28,43^, in which chirality is introduced as an isotropic magnetoelectric coupling. The constitutive relations,

2 $${{\bf{D}}}=\varepsilon {{\bf{E}}}+{{\rm{i}}}\xi {{\bf{H}}},$$

3 $${{\bf{B}}}=\mu {{\bf{H}}}-{{\rm{i}}}\xi {{\bf{E}}},$$

where **E** and **H** are the electric and magnetic fields, **D** and **B** are the electric displacement and magnetic induction, $\varepsilon$ and $\mu$ are the permittivity and permeability of the medium, and $\xi$ is the Pasteur (chirality) parameter. The parameter is a pseudoscalar and therefore changes sign between enantiomers. In this bulk-constitutive picture, chirality enters only through $\xi$ and does not explicitly encode effects that depend on the charge state or orientational polarisation of an interfacial molecular layer.

**Fig. 6:**
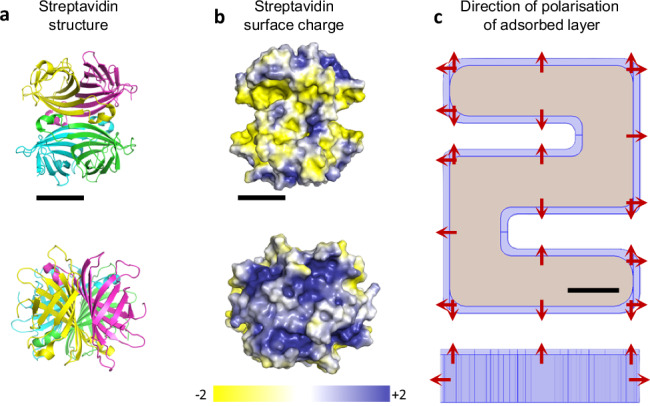
Chiral interfacial layer underpinning boundary-condition coupling. **a** Protein structures (scale bar: 2 nm). **b** Electrostatic surface potential maps showing heterogeneous charge distributions that, upon adsorption with hydration and counter-ions, constitute a chiral, polarisable interfacial layer (scale bar: 2 nm). The colour bar shows surface charge. **c** Schematic showing how this interfacial region modifies electromagnetic boundary conditions at the surface of an enantiomorphic silicon resonator, enabling chirality transmission across length scales without invoking molecular alignment (scale bar: 150 nm).

An alternative model: Protein binding produces (Fig. 6a–c) a structured interfacial region at the silicon–solution boundary comprising the (surface) charged proteins, their tightly associated hydration shell, and the equilibrium distribution of counter-ions. The term ‘ionic conductor’ is used here in a quasi-static sense: counter-ions are mobile on experimental timescales and equilibrate around the protein, establishing a structured space-charge and screening environment at the interface. At optical frequencies, mobile ions cannot follow the field cycle-by-cycle. Instead, consistent with interfacial electrodynamics formalisms, the optical consequences of interfacial charge are most naturally captured through modified electromagnetic boundary conditions rather than through bulk constitutive relations^44^.

The interfacial layer is therefore described by an effective complex surface response that combines the optical-frequency response of bound partial charges within the protein with a screened interfacial admittance associated with the ionic environment. Although the interface is electrically neutral overall, it contains a spatially heterogeneous distribution of bound charge defined by the protein’s higher-order structure, together with counter-ions that determine the local screening environment. The chirality of this interfacial layer is set by molecular stereostructure and does not depend on the sign of the net protein charge.

The blue shift: The bound-charge component of the interfacial layer generates an effective surface polarisation that enters the normal electromagnetic boundary condition^16,17^. We write this as

4 $${{{\bf{E}}}}_{{{\rm{out}}}}^{\perp }-{{{\bf{E}}}}_{{{\rm{in}}}}^{\perp }=-\frac{{P}_{{{\rm{n}}}}}{{\varepsilon }_{0}},$$

where ${{{\bf{E}}}}_{{{\rm{out}}}}^{\perp }$ and ${{{\bf{E}}}}_{{{\rm{in}}}}^{\perp }$ are the electric fields, normal to the surface outside and inside the silicon nanostructure, respectively, and ${P}_{{{\rm{n}}}}$ is an effective surface polarisation density representing the spatially averaged optical-frequency response of the interfacial polarisation layer under strong hydration and ionic screening. Importantly, in the present system, ${P}_{{{\rm{n}}}}$ is an effective, screened interfacial polarisation density: it is not a microscopic protein dipole moment, and it does not assume a single well defined protein orientation, which is not expected for glutaraldehyde coupling. Because this is an effective, optically driven interfacial polarisation under ionic screening, its contribution to resonance shifting is not expected to change sign simply with reversal of the protein’s net charge.

Equation (4) weakens the effective dielectric contrast at the silicon interface by reducing the normal displacement-field matching. This boundary-condition weakening can shift dielectric resonances to shorter wavelengths, producing a blue shift rather than the red shift expected from bulk refractive-index loading^18,45^. We therefore interpret the resonance blue shift as a boundary-condition effect of an interfacial polarisation layer, not as a conventional dielectric overlayer response.

Meta-diastereomer formation: The distinguishing feature of a meta-diastereomer is that the coupled optical state depends on the relative handedness of the molecular layer and the chiral electromagnetic environment. Experimentally, this is reported most directly by the emergence of a handedness-dependent divergence in reflectance-derived linear dichroism. While mobile counter-ions do not respond directly at optical frequencies, they define the static screening environment and therefore contribute to the effective surface admittance of the interfacial layer. The spatial arrangement of bound charges and counter-ions around a chiral protein is itself chiral, which gives the interface a pseudoscalar surface response and modify the boundary condition for the tangential component of the field. A minimal phenomenological description consistent with chiral interface models for thin chiral systems can be written as^46,47^

5 $${{{\bf{J}}}}_{s}={\sigma }_{0}(\omega ){{{\bf{E}}}}_{\parallel }+{\sigma }_{\chi }(\omega )(\hat{{{\bf{n}}}}\times {{{\bf{E}}}}_{\parallel }),$$

where ${\sigma }_{0}\left(\omega \right)$ is the achiral component of an effective surface admittance (capturing screened interfacial response), ${\sigma }_{\chi }(\omega )$ is a chiral pseudoscalar term, ${{{\bf{E}}}}_{\parallel }$ is the tangential electric field, and $\hat{{{\bf{n}}}}$ is the surface normal. The term $\left(\hat{{{\bf{n}}}},|,{{{\bf{E}}}}_{\parallel }\right)$ represents a ${90}^{\circ }$ in-plane rotation of ${{{\bf{E}}}}_{\parallel }$ and therefore encodes the pseudoscalar symmetry required for a chiral surface response.

The meta-diastereomeric character of the coupled system follows directly from Eq. (5): when a chiral interfacial environment (${\sigma }_{\chi }\ne 0$), mirror-related S_LH_ and S_RH_ resonators are no longer constrained to remain mirror equivalent. Because the S-shaped resonators generate different tangential near-field distributions for orthogonal linear polarisations, the ${\sigma }_{\chi }$ term perturbs the boundary conditions asymmetrically. Crucially, this perturbation projects differently onto the mirror-related field patterns of S_LH_ and S_RH_, yielding two symmetry-inequivalent coupled states (meta-diastereomers) whose difference is reported directly by the handedness-dependent linear dichroism.

A bulk Pasteur description treats the protein layer as a homogeneous chiral dielectric in which chirality enters via magnetoelectric coupling in the constitutive relations and accumulates through propagation. For a 10–20 nm protein film (*t* ≪ *λ*), any propagation-based bulk chiral response enters the scattering problem only as a weak perturbation. Moreover, bulk optical activity primarily produces helicity-dependent phase and polarisation rotation, whereas the key experimental observable here is a handedness-dependent divergence in a linear-dichroic quantity.

In contrast to the Pasteur description, Eqs. (4) and (5) describe meta-diastereomer formation through direct perturbation of the boundary conditions that determine the silicon resonances. Equation (4) modifies the boundary condition for the normal field and can therefore dominate the sign of the spectral shift. Equation (5) modifies the boundary condition for the tangential field through a pseudoscalar chiral operator and provides a direct symmetry route by which molecular stereostructure is encoded into a linear optical observable. In the simulations below, we therefore compare a Pasteur-only overlayer with the same overlayer supplemented by the interfacial polarisation term, to test which ingredients are required to reproduce the observed blue shift and handedness-dependent linear dichroism.

To clarify the origin of the observed linear dichroism, it is essential to distinguish between structural anisotropy of the metasurface and the protein-induced interfacial response. Linear dichroism requires two inequivalent linear excitation channels, which in the present system are provided by the intrinsic anisotropy of the S-shaped metasurface modes. This anisotropy exists prior to adsorption and gives rise to finite LD in buffer, while mirror-related ${{{\rm{S}}}}_{{{\rm{LH}}}}$ and ${{{\rm{S}}}}_{{{\rm{RH}}}}$ structures remain LD-identical by symmetry.

Protein adsorption introduces an interfacial ionic layer that modifies the tangential electromagnetic boundary conditions through an effective surface current response. This layer is not assumed to possess intrinsic in-plane (*x*–*y*) anisotropy or preferential molecular alignment. Rather, its role is to perturb the coupling between the incident field and the anisotropic metasurface eigenmodes. Because the near-field distributions associated with orthogonal linear polarisations differ in amplitude, phase and spatial structure, a single interfacial perturbation produces unequal effective damping and phase shifts for the two excitation channels, thereby modifying the measured LD.

Within this framework, chirality enters through an antisymmetric in-plane surface response that mixes orthogonal tangential field components. In an anisotropic resonant system, such a helicity-dependent perturbation does not solely generate circular dichroism but also alters the relative extinction of linearly polarised modes. Importantly, the projections of this chiral interfacial response onto the metasurface eigenmodes are mirror-inverted for ${{{\rm{S}}}}_{{{\rm{LH}}}}$ and ${{{\rm{S}}}}_{{{\rm{RH}}}}$ geometries, breaking their equivalence after adsorption and giving rise to handedness-dependent changes in LD.

Any effective in-plane anisotropy of the protein-modified interface should therefore be understood as an emergent property of the coupled metasurface–ionic system, rather than as a pre-existing anisotropy of the ionic layer itself. A formal Jones–Müller analysis demonstrating the linear sensitivity of LD to a chiral diagonal perturbation is provided in Supplementary Table 1^48^.

Electromagnetic numerical modelling: To test whether the experimental signatures can be reproduced by a bulk chiral overlayer alone, or whether an additional interfacial boundary perturbation is required, we performed finite-element simulations in COMSOL Multiphysics.

As an internal check, we first modelled the refractive-index control (PBS versus rac-2-butanol), which reproduces the observed red shift (Supplementary Fig. 3a). Consistent with experiment (Supplementary Fig. 1), introducing a Pasteur chiral dielectric response in the surrounding medium does not generate handedness-dependent differences between mirror-equivalent S_LH_ and S_RH_ under linearly polarised excitation (Supplementary Fig. 3b).

The protein layer is represented as a thin dielectric slab adjacent to the silicon surface with chiral response described by a Pasteur coefficient, *ξ*, using parameter values established in previous numerical treatments of biomolecular layers in nanophotonic environments. In addition, we include an effective surface polarisation normal to the silicon surface. This contribution enters explicitly through the boundary-condition term ${P}_{{{\rm{n}}}}$ in Eq. (4), which modifies the boundary condition for the normal component of the displacement-field at the interface.

Within this phenomenological description, the simulated resonant modes exhibit a blue shift consistent in sign and magnitude with experiment is obtained for ${P}_{{{\rm{n}}}}=1\times {10}^{-8}\,{{\rm{C}}}\,{{{\rm{m}}}}^{-2}$ (Supplementary Figs. 3c, 14). This value is approximately four orders of magnitude smaller than estimates for an isolated protein molecule, consistent with reduction of the effective interfacial polarisation by Debye screening and orientational averaging at the surface. Accordingly, ${P}_{{{\rm{n}}}}$ should be interpreted as a macroscopic effective boundary term, not as a microscopic molecular dipole density.

**Fig. 7:**
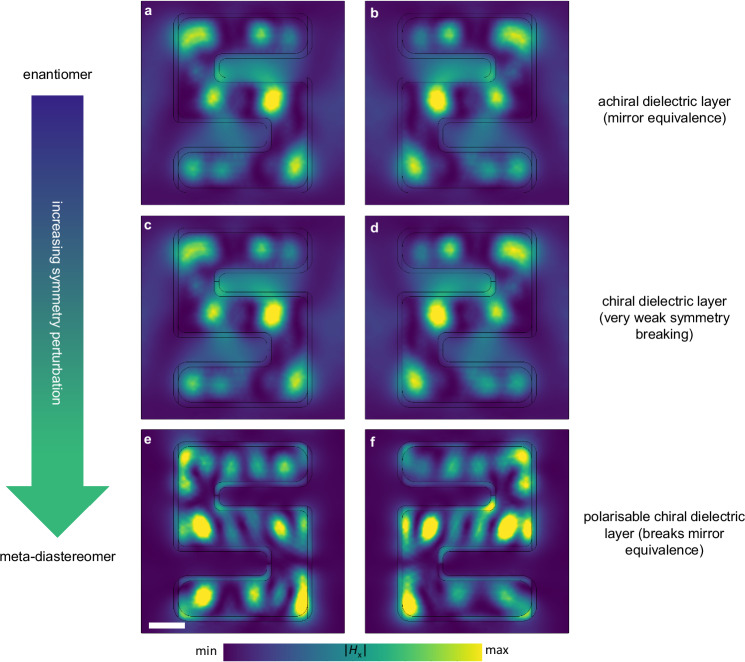
Boundary-condition-driven symmetry breaking and meta-diastereomer formation. Simulated near-field |*H*_*x*_| distributions for mirror-equivalent S_LH_ and S_RH_ under progressively increasing perturbation by an (**a**, **b**) achiral dielectric, (**c**, **d**) chiral dielectric and (**e**, **f**) chiral, polarisable interfacial layer. Simulation preformed at 650 nm and with the polarisation directed away from the surface. Maps for the polarisation point towards the surface can be found in Supplementary Fig. 15. The arrow indicates increasing interfacial perturbation strength. While the nanostructure geometry remains unchanged, modification of the electromagnetic boundary conditions redistributes the resonant modes asymmetrically, breaking mirror equivalence and generating symmetry-inequivalent electromagnetic responses characteristic of meta-diastereomer formation. The scale bar is 150 nm.

The consequences of this interfacial perturbation are directly visualised in the simulated field distributions. Figure 7 shows the magnitude of the magnetic-field component ${H}_{x}$ in the plane of the silicon metasurfaces, for the position of the dipole layer pointing away from the surface. Similar maps are obtained for the inward pointing polarisation, indicating the effects are not dependent on the sign of the polarisation (Supplementary Fig. 15). When only a (Pasteur) chiral dielectric layer is introduced, the mirror equivalence of the near fields of S_LH_ and S_RH_ are not strongly perturbed. The two metasurfaces therefore remain electromagnetically enantiomeric.

In contrast, introduction of a ‘polarised’ chiral interfacial layer produces asymmetric changes in the field intensities for S_LH_ and S_RH_ structures. While the spatial arrangement of the fields remains unchanged, their magnitudes differ between the two handednesses. As a result, the S_LH_ and S_RH_ systems are no longer related by mirror symmetry, despite both remaining chiral. In electromagnetic terms, the two interfaces therefore form a diastereomeric pair. This symmetry reduction provides the symmetry route required for the experimentally observed handedness-dependent linear dichroism

Importantly, field maps taken in a mid-plane cut through the structure show a weaker asymmetry (Supplementary Fig. 16), consistent with the perturbation being localised to the silicon–biomolecule interface rather than arising from a bulk chiral response throughout the surrounding medium.

The same framework naturally accounts for the sensitivity of the meta-diastereomer response to protein structure. The effective interfacial polarisation is expected to depend on the degree of orientational and conformational order within the protein layer. Increased structural order leads to a larger net polarisation and enhanced asymmetry between S_LH_ and S_RH_ responses, whereas structural disorder suppresses the effective interfacial polarisation and reduces the meta-diastereomeric contrast.

This effect is particularly relevant for polyclonal antibodies such as anti-strept, which exhibit substantial conformational flexibility and a wide distribution of binding geometries arising from multiple epitopes. Such structural heterogeneity is expected to reduce the net interfacial polarisation and therefore weaken the meta-diastereomeric response, consistent with the reduced asymmetry observed experimentally relative to strept.

In summary, we demonstrate the formation of meta-diastereomers: symmetry-inequivalent hybrid states that arise when molecular stereostructure couples to enantiomorphic dielectric metasurfaces. This coupling is driven by adsorption-induced modification of electromagnetic boundary conditions, where chirality is conveyed by a thin, polarisable interfacial layer that breaks mirror equivalence at the solid–liquid boundary. By rendering linear optical responses stereostructurally informative, this mechanism departs fundamentally from the established reliance on “superchiral” field enhancements.

Benchmarking this effect clarifies its distinct physical origin compared to established nanophotonic phenomena. Unlike refractive-index loading, which responds to isotropic permittivity changes in the bulk, meta-diastereomer formation depends on anisotropic interfacial charge organisation. Furthermore, unlike superchiral engineering^21,24,32–34,49^, which imposes strict geometric constraints to amplify weak CD responses, meta-diastereomers do not amplify intrinsic molecular CD. Instead, the interface itself converts stereochemical organisation directly into symmetry-inequivalent optical states under orthogonal linear polarisations.

Crucially, the metasurface supports polarisation-dependent tangential near-field distributions that couple differently to the chiral surface term. The resulting handedness-dependent linear dichroism tracks the stereostructural organisation of the molecular layer and is suppressed by orientational averaging. Together, these results establish boundary-condition-mediated chirality transfer as a design principle for dielectric nanophotonics, shifting the focus from chiral-field optimisation to the control of interfacial perturbations where molecular and photonic length scales intersect.

## Methods

### Materials

Phosphate buffered saline solution of pH 7.4 (10010031) was purchased from Gibco. Sodium acetate buffer solution of pH 5.2 (S7899) was purchased from Merck. (S)-(+)−2-butanol (4221-99−2), (R)-(−)-2-butanol (14898-79-4) and (+/−)−2-butanol were purchased from Thermo Scientific. Glutaraldehyde solution (Grade I, 50% in H_2_O) was purchased from Merck (G7651). Poly-L-lysine solution (0.01%) was purchased from Merck (A-005-C). Streptavidin (Q10121MP) was purchased from Thermo Fisher. Rabbit streptavidin antibody (200-401-095) was purchased from Rockland.

### Sample fabrication

Sample fabrication was performed at the James Watt Nanofabrication Centre (JWNC). The S-structures were created using electron-beam lithography. Quartz glass slides were first cleaned by ultrasonic agitation using an AMI cleaning sequence (acetone, methanol, and isopropyl alcohol for 5 min each). Following cleaning, the slides were dried under a nitrogen flow and subjected to an oxygen plasma treatment for 5 min at 100 W. Amorphous silicon was then deposited onto the substrates using plasma-enhanced chemical vapor deposition (PECVD) with an SPTS Delta tool. The samples were subsequently cleaned again, but with a 1 min low-power (60 W) plasma treatment.

Next, a bilayer of PMMA resist was spun onto the substrates at 4000 rpm for 1 min and baked at 180 °C for 5 min between each spin. A 10 nm aluminium conducting layer was deposited using a PLASSYS MEB 550 s evaporator. Patterns were designed using L-Edit CAD software and written with a Raith EBPG 5200 electron beam lithography system operating at 100 kV. The aluminium was removed using CD−26, and the resist was developed in a 3:1 mixture of MIBK: IPA at 23.2 °C for 1 min, then rinsed in IPA for 5 s and deionised water before being dried under nitrogen flow. A 50 nm nichrome etch mask was then deposited on the sample. This was followed by a lift-off procedure in acetone at 50 °C overnight, with the sample agitated to remove any residual resist and excess metal. The silicon was etched using an STS tool with a custom recipe of C4F8 / SF6 (90/30 cm^3^ STP min^−1^), 600 W, 9.8 mTorr, 20 °C for 3 min. The NiCr etch mask was removed by immersion in chromium etchant and 60% nitric acid. The final steps involved an AMI cleaning sequence and low-power plasma cleaning.

### Surface functionalisation with biomolecules

Freshly fabricated metasurfaces were used for all experiments. Samples were first equilibrated in PBS for ≥5 min. A PLL (1:100 in H_2_O) pre-coating was applied to promote biomolecular adhesion by incubating the samples in PLL solution for 15 min, followed by thorough rinsing with PBS. The glutaraldehyde solution (50% in H_2_O) was diluted to 12.5% and was then introduced and incubated for 45 min to generate aldehyde-terminated binding sites, after which the samples were extensively rinsed with PBS.

Strept- or anti-strep- were subsequently added and incubated for 30 min, allowing covalent attachment via glutaraldehyde-mediated cross-linking. Unbound material was removed by rinsing with PBS. Residual reactive aldehyde groups were passivated by incubation with 40 mM ethanolamine for 20 min, followed by rinsing and exchange into fresh PBS prior to optical measurements.

For experiments involving protein–protein interactions, a final binding step was performed by incubating strept-functionalised samples with anti-streptavidin, or anti-strept-functionalised samples with streptavidin, for 1 h. In the case of anti-strept binding this was followed by a wash with PBS. While for strept binding to anti-strept, LD^R^ data were collected before and after PBS wash.

### Reflectance measurements

The substrates were placed in a custom-designed sample holder, sealed with a FastWell silicone gasket and covered with a clear borosilicate glass slide. The nanoarrays were immersed in PBS by injecting the buffer into the cavity of the holder. The sample was mounted on the stage of a custom-built Stokes polarimeter. Incident polarisation was adjusted using a linear polariser, and optical rotation dispersion (ORD) was measured by capturing intensities at four analyser angles: 0°, 45°, 90°, and 135°. Reflectance data were recorded with the analyser set at 0°.

### Müller matrix polarimetry

For MMP measurements, samples were prepared in the same manner as for Stokes polarimetry. The highly collimated beam of B23 beamline at Diamond Light Source enabled the use of the MMP that consist of two pairs of photoelastic modulators that flanked the sample, serving as the polarisation state generator and analyser^50^. This setup enables the production and measurement of all required light states to determine the differential 16 Müller matrix elements. The LD’, LD”, LB’, LB”, CD, CB, Abs, and *g*-factor were calculated using the Analytic Inversion method, and for the DI according to Gill and Bernabeu equation^51^ with *m* the elements of the differential Müller matrix.

6 $${{\rm{DI}}}=\sqrt{\frac{1}{3{m}_{00}^{2}}\left({\sum }_{i,j=0}^{3}{m}_{{ij}}^{2}-{m}_{00}^{2}\right)}.\,$$

### Calculation of protein electrostatic potentials

The surface charge densities were calculated using the crystal structures of streptavidin. The coordinates of protein were generated and all amino acid sidechains were incorporated. The N-terminal histidine tag was omitted from the models as this is expected to be disordered and its effect should be consistent for both proteins. Models with calculated hydrogen positions and the partial charges were produced using PROPKA 3.0 software package (can find the ionisable group of the protein and predict their behaviour) for each of the pH environments considered. These models were then used to calculate surface charges using the open-source Adaptive Poisson-Boltzmann Solver (APBS) electrostatic calculation program. The surface charge densities were displayed on a molecular surface using the PYMOL visualization software.

### Numerical simulations

Simulations were conducted using the commercial finite element analysis software COMSOL Multiphysics v6.3, specifically the Wave Optics module. The nanostructure was modelled within a cuboid unit cell, where the *x* and *y* dimensions defined the metamaterial’s periodicity as determined from AFM images. The *z* dimensions of the cell were sufficiently large (≥*λ*_max/2_) to ensure that near fields from the nanostructures did not interact with integration surfaces above the structure; the total height of the cell was set to 1800 nm. The unit cell was divided into domains of different thicknesses, with the top and bottom 200 nm domains serving as perfectly matched layers (PMLs) to absorb all reflections. The boundary adjacent to the upper PML acted as the excitation port, from which the incident light was directed and its polarization specified. Reflection intensity was also calculated at this port. The silicon structure was situated near the centre of the cuboid, while the boundary adjacent to the lower PML functioned as the outgoing port.

To model the racemic-site (RS) array, a repeating unit of the RS array containing two RH and two LH structures was used. The width and depth of the model were therefore doubled, with the height remaining the same. To validate the model, these simulations were also performed for 4xLH and 4xRH structures to compare against single-structure simulations.

### Statistics and reproducibility

The MMP measurements were performed on two different samples, with each measurement repeated twice. Reflectance data was an average of either two or five independent measurements from two samples. LD^R^ measurements were performed on two samples with each measurement repeated five times. The data presented in this manuscript represents the average of these two and five measurements from a single representative sample, respectively.

The MMP CD data have been smoothed using smoothed 20-point Savitzky-Golay method and the raw data are provided as Supplementary Data 1.

### Reporting summary

Further information on research design is available in the Nature Portfolio Reporting Summary linked to this article.

## Supplementary information

Supplementary information

Description of Additional Supplementary Files

Supplementary Data 1 (Raw data)

Reporting Summary

Transparent Peer Review file

## Source data

Source data

## Data Availability

The data that support the findings of this study are available from the corresponding authors upon request. Unprocessed raw data are available as Supplementary Data 1. Source data are provided with this paper.
