# Supplementary material for: Metasurface analogues of molecular diastereomers from hierarchical multiscale chiral interactions with biomolecules
Source: Nat Commun. 2026 Apr 22;17:5541. doi: 10.1038/s41467-026-72200-6 (PMC13287669; doi:10.1038/s41467-026-72200-6)
Supplement: Supplementary file 1 — Supplementary information [file 41467_2026_72200_MOESM1_ESM.pdf]

Supplementary information for

Metasurface analogues of molecular diastereomers from hierarchical multiscale chiral interactions with biomolecules

Dominic J.P. Koyroytsaltis-McQuire[1], Shailendra K. Chaubey[1*], Rahul Kumar[1], Paula L. Lalaguna[1], Tamas Javorfi[2], Giuliano Siligardi[2], Affar Karimullah[1], Adrian J. Lapthorn[1], Yoshito Y. Tanaka[3], Shun Hashiyada[3,4], Nikolaj Gadegaard[5], Malcolm Kadodwala[1*]

[1] School of Chemistry, University of Glasgow, Glasgow, G12 8QQ, UK.

[2] Diamond Light Source Ltd., Harwell Science and Innovation Campus, Didcot, OX11 0DE, UK.

[3] Research Institute for Electronic Science, Hokkaido University, Sapporo, Hokkaido 001-0021, Japan.

[4] PRESTO, Japan Science and Technology Agency, Kawaguchi, Saitama, 332-0012, Japan.

[5] School of Engineering, Rankine Building, University of Glasgow, Glasgow G12 8LT, UK.

* Corresponding Authors

E-mail: Malcolm.kadodwala@glasgow.ac.uk,

shailendrakumar.chaubey@glasgow.ac.uk

1. Enantiomer dependence of magnetic dipole resonance

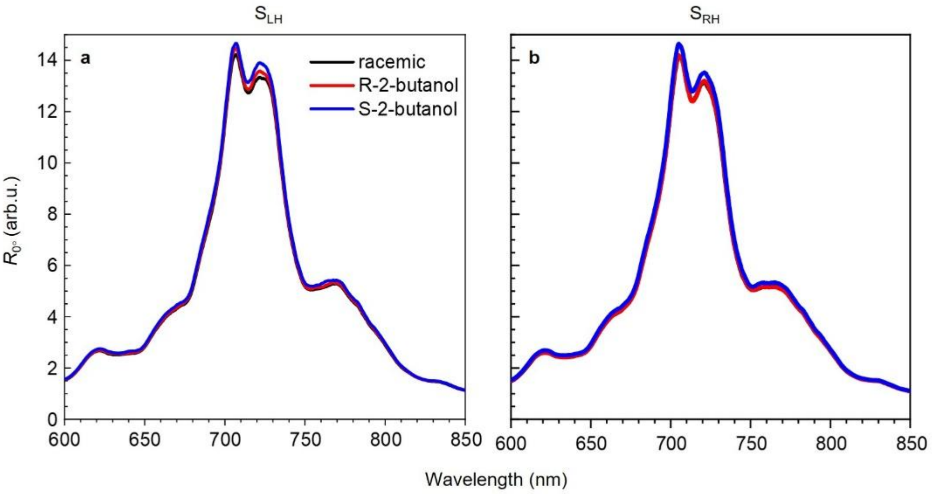

Supplementary Fig. 1 Enantiomer insensitivity of S nanostructures.

Reflectance spectra from **a** S_{LH} and **b** S_{RH} for Racemic (black), R (red)- and S (blue)-2-butanol. The position of the magnetic dipole resonance is insensitive to the chirality of the liquid.

2. Racemic data

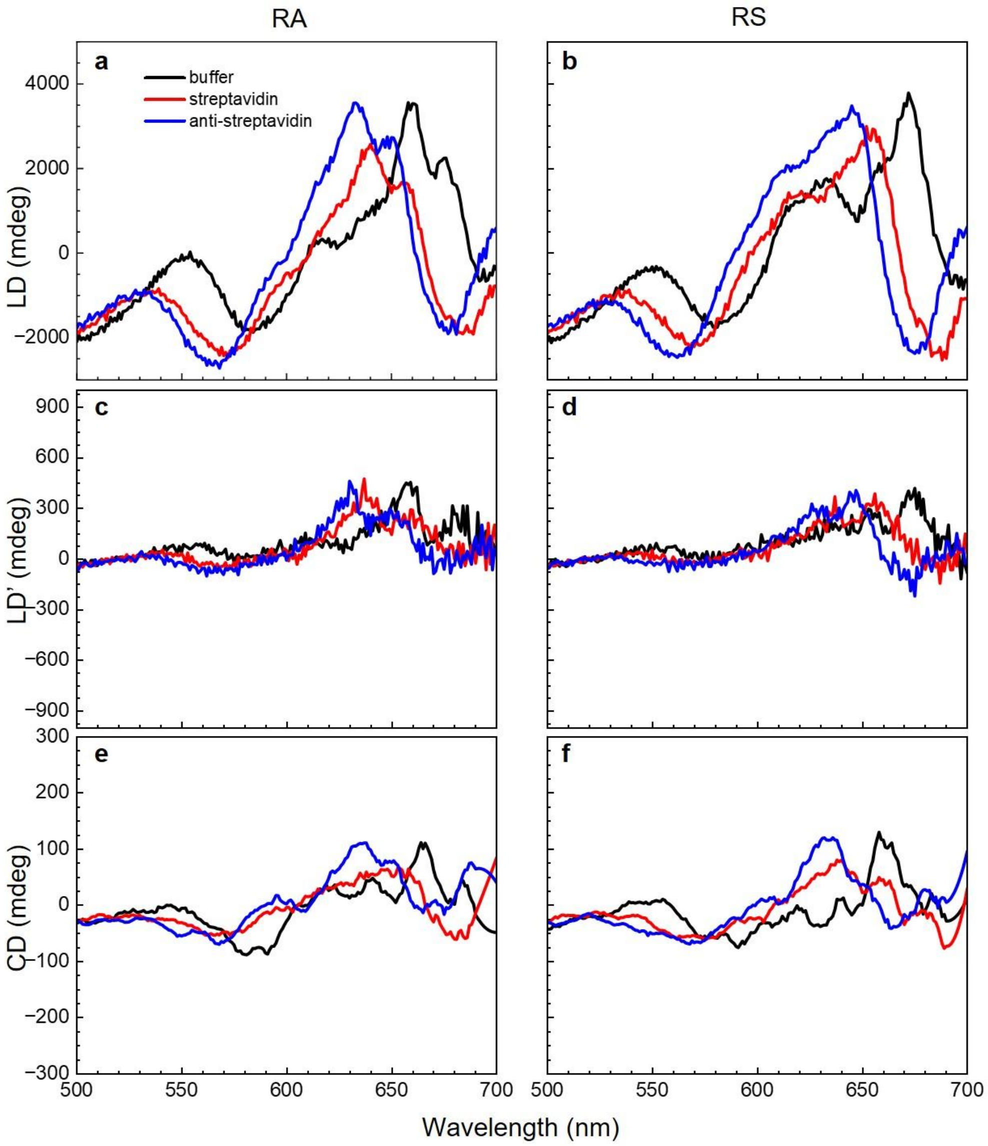

Supplementary Fig. 2 MMP data for racemic metasurfaces.

LD, LD' and CD MMP data for the racemic arrays of the ~210 nm sample. LD data for the **a** RA and **b** RS arrays is shown for the sample immersed in PBS with no biomolecule present (black) and with streptavidin (red) and antistrep (blue) deposited. Equivalent data is presented for **c**-**d** LD' and **e**-**f** CD. Note the scales for LD' and CD are reduced compared to the enantiomorphic data. The CD data is smoothed as before.

3. Numerical Simulation of blue shift

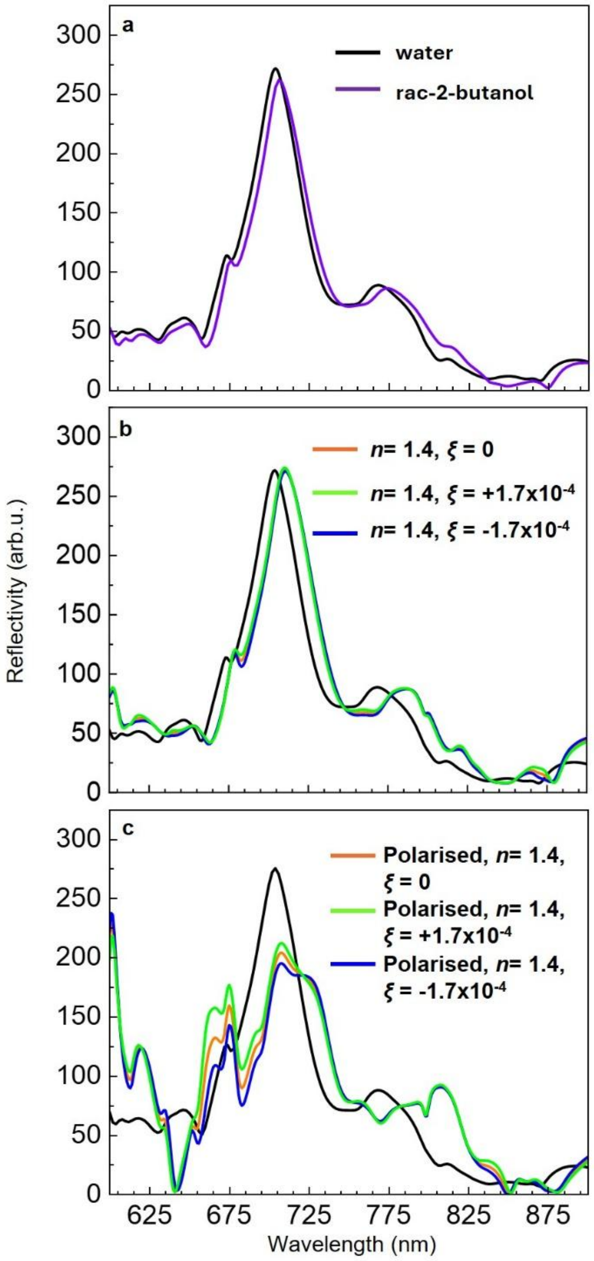

Supplementary Fig. 3 Numerical simulations replicate blue shift.

Numerical simulations for: **a** an LH structure immersed in water and rac-2-butanol; **b** an LH structure immersed in buffer with a chiral dielectric layer; and **c** an LH structure immersed in buffer with a polarized chiral dielectric layer. The simulations with a negative Pasteur coefficient (ξ) are symmetry equivalent to an RH structure with a positive ξ. n is the real part of the refractive index.

4. AFM data

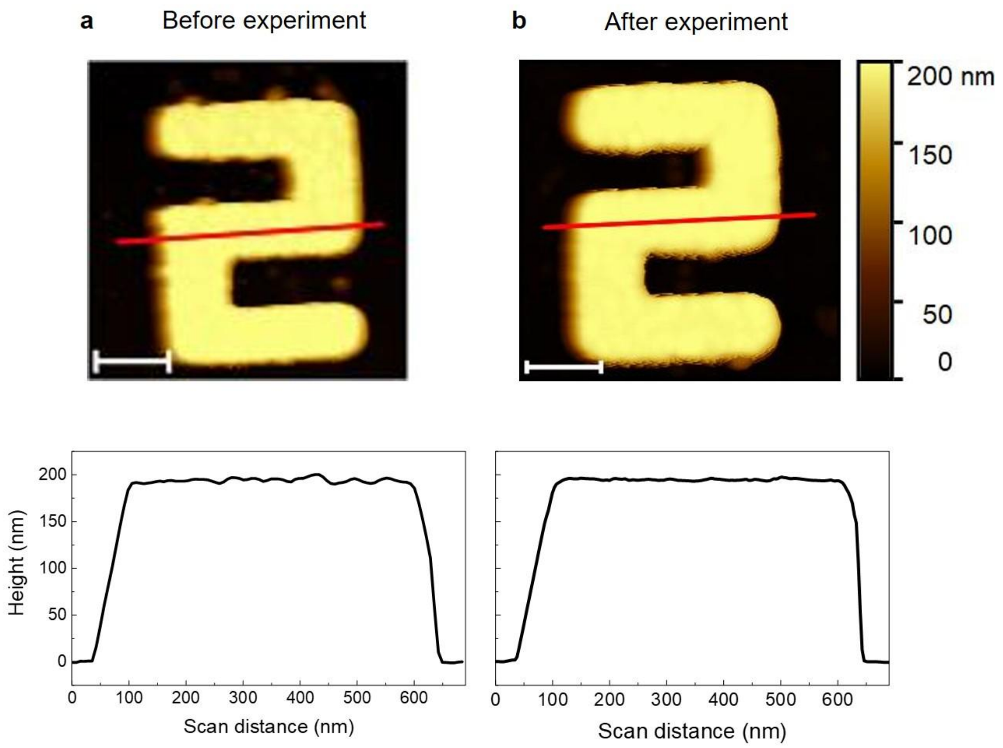

Supplementary Fig. 4 AFM data show no morphological change after protein immobilisation.

AFM data collected for the sample used after streptavidin immobilisation. **a** AFM image of a pristine LH structure prior to deposition, and the associated height profile extracted from the red line. **b** Equivalent data after experiment, following the deposition of antistrep onto the strept functionalised surface. The scale bar is 200 nm.

5. Müller matrix polarimetry

M_{00} T	M_{01} -LD	M_{02} -LD′	M_{03} CD
M_{10} -LD	M_{11} T	M_{12} CB	M_{13} -LB′
M_{20} -LD′	M_{21} -CB	M_{22} T	M_{23} LB
M_{30} CD	M_{31} LB′	M_{32} -LB	M_{33} T

Supplementary Table 1. Elements of the Müller matrix in MMP.

6. Müller matrix for RA structure in Buffer

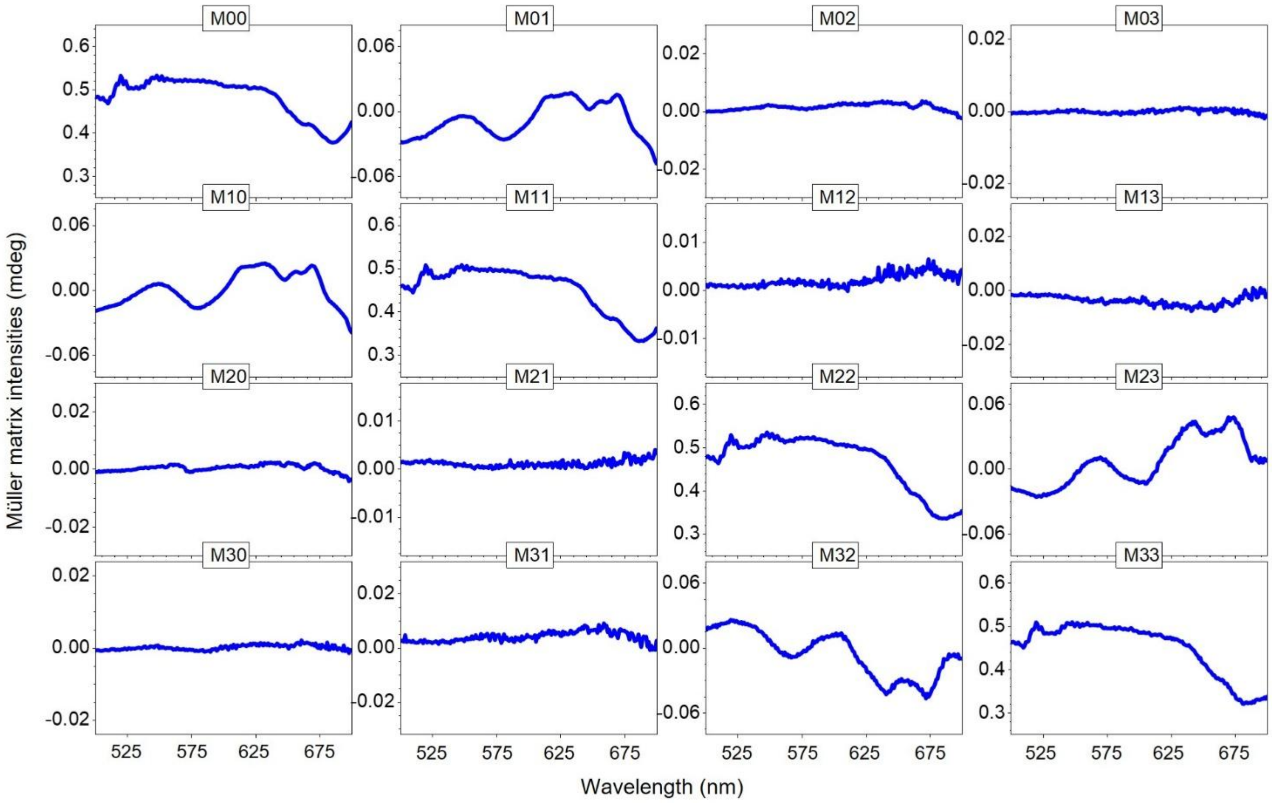

Supplementary Fig. 5 Müller matrix for RA in buffer.

All the MMP elements for the RA metamaterial in buffer before the mobilisation of streptavidin.

7. Müller matrix for RS structure in Buffer

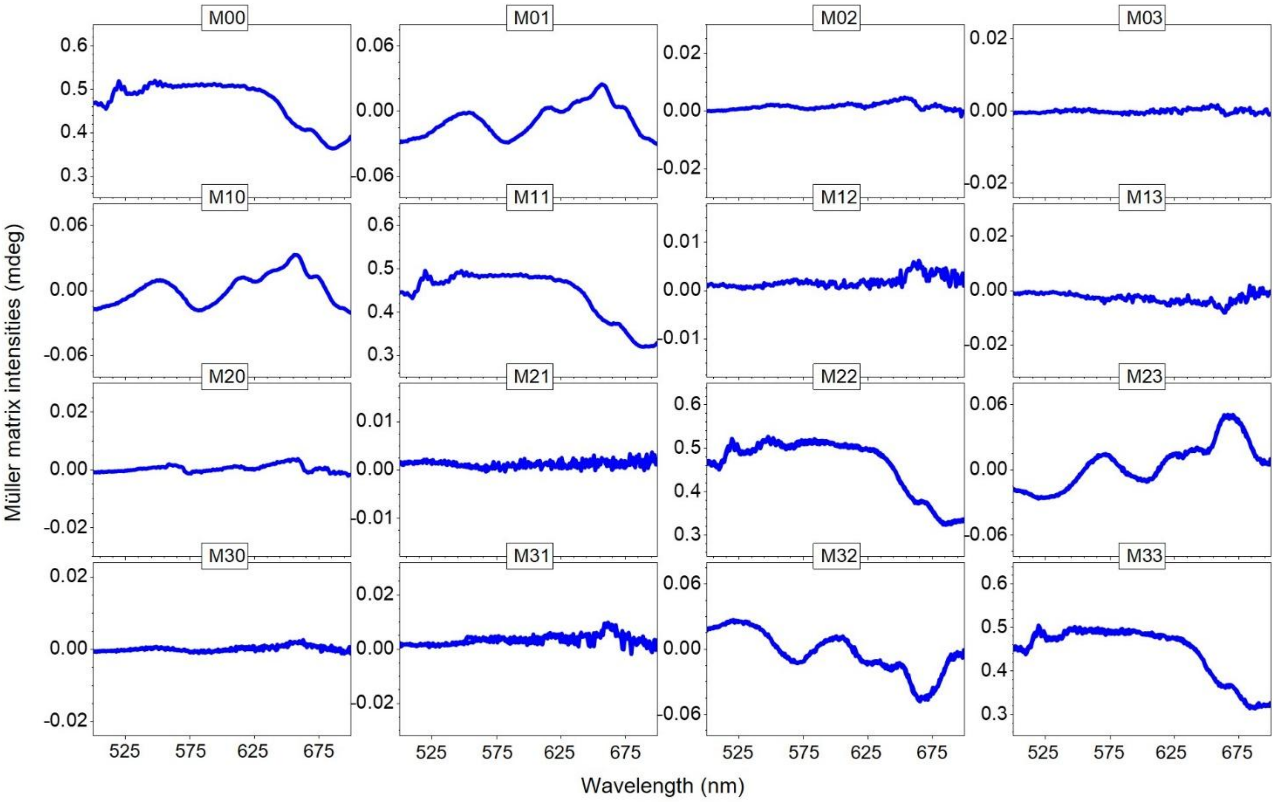

Supplementary Fig. 6 Müller matrix for RS in buffer.

All the MMP elements for the RS metamaterial in buffer before the mobilisation of streptavidin.

8. Müller matrix for RA structure with Streptavidin

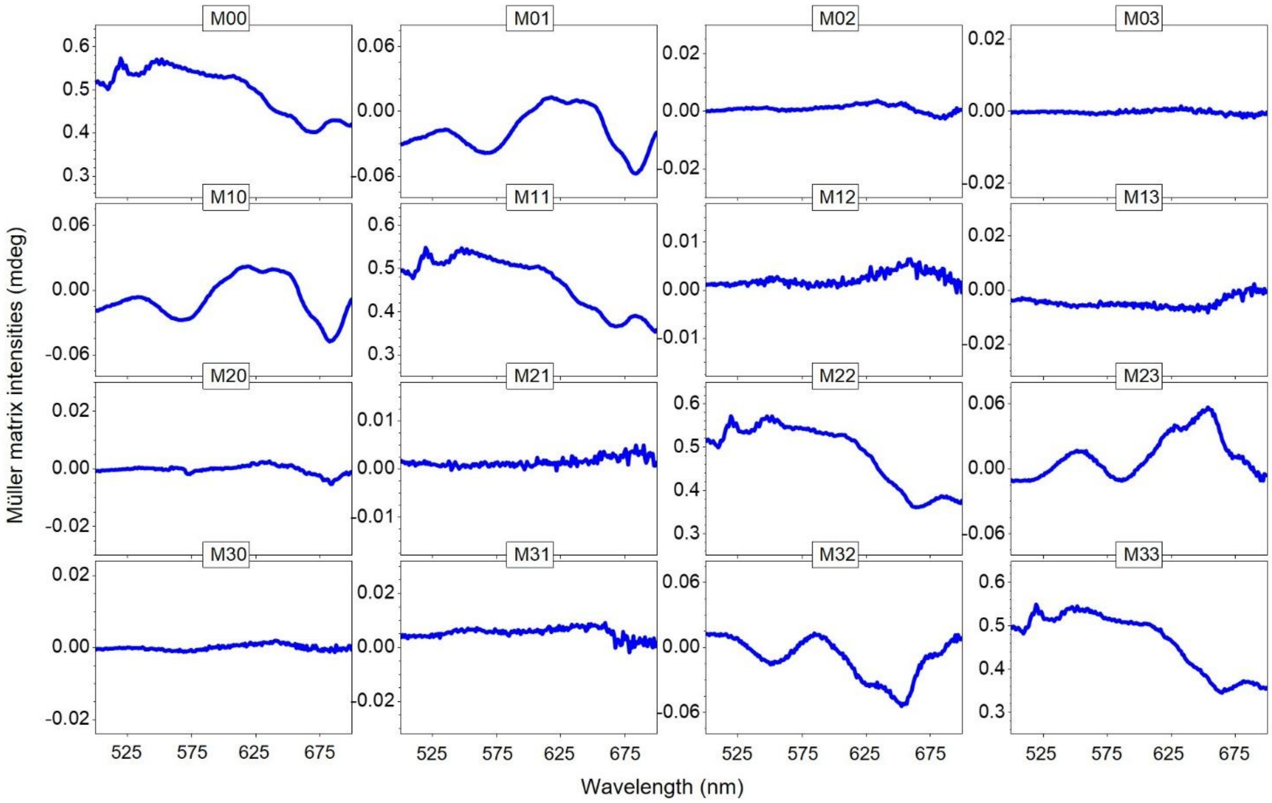

Supplementary Fig. 7 Müller matrix for RA with streptavidin.

All the MMP elements for the RA metamaterial in the buffer after the mobilisation of streptavidin on metamaterial.

9. Müller matrix for RS structure with streptavidin

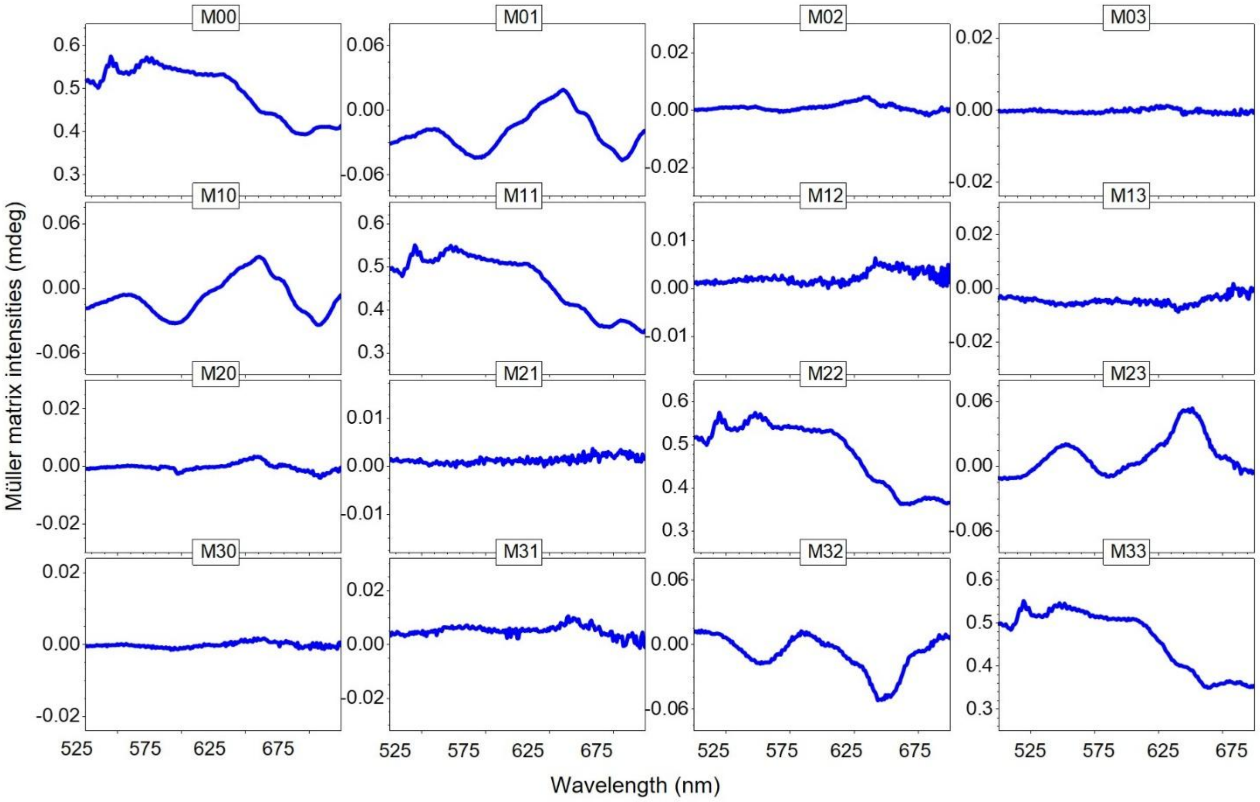

Supplementary Fig. 8 Müller matrix for RS with streptavidin.

All the MMP elements for the RS metamaterial in the buffer after the mobilisation of streptavidin on metamaterial.

10. Müller matrix for RA structure with anti-streptavidin

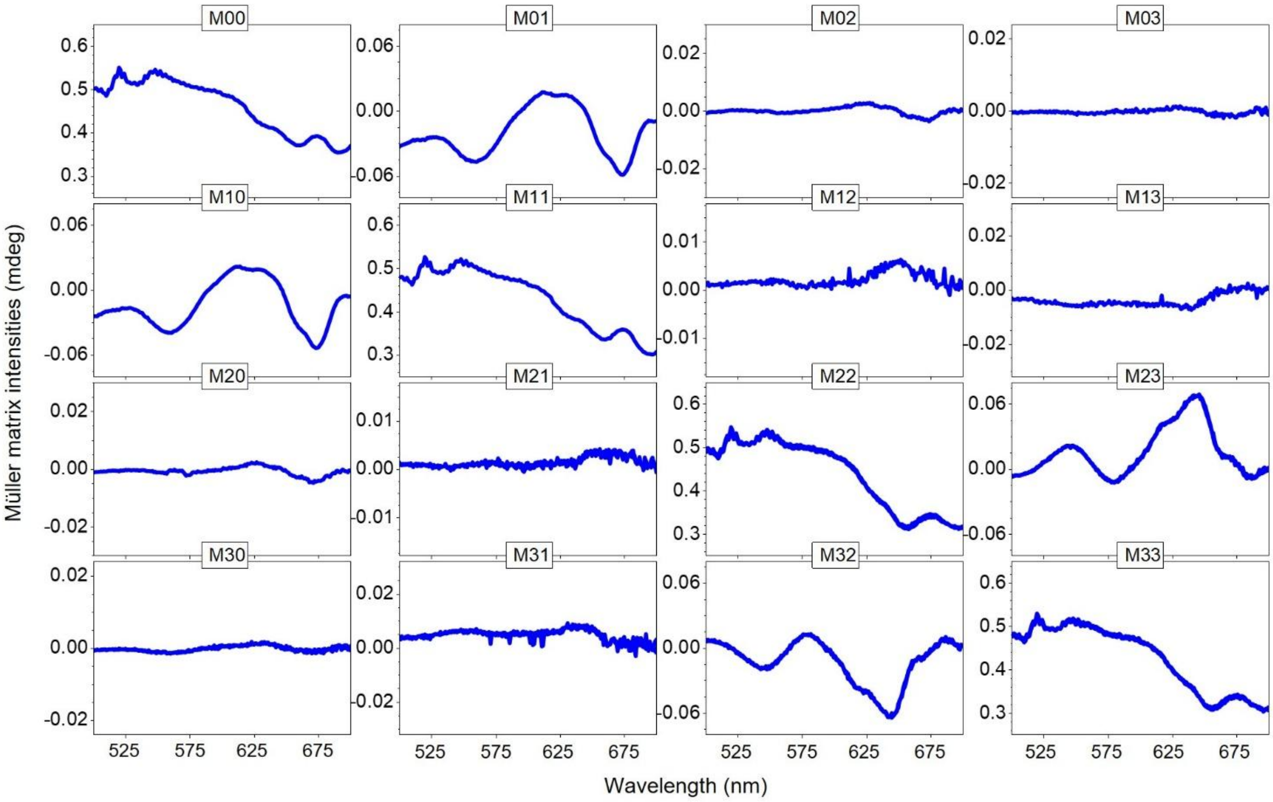

Supplementary Fig. 9 Müller matrix for RA with anti-streptavidin.

All the MMP elements for the RA metamaterial in the buffer after the mobilisation of anti-streptavidin on metamaterial.

11. Müller matrix for RS structure with anti-streptavidin

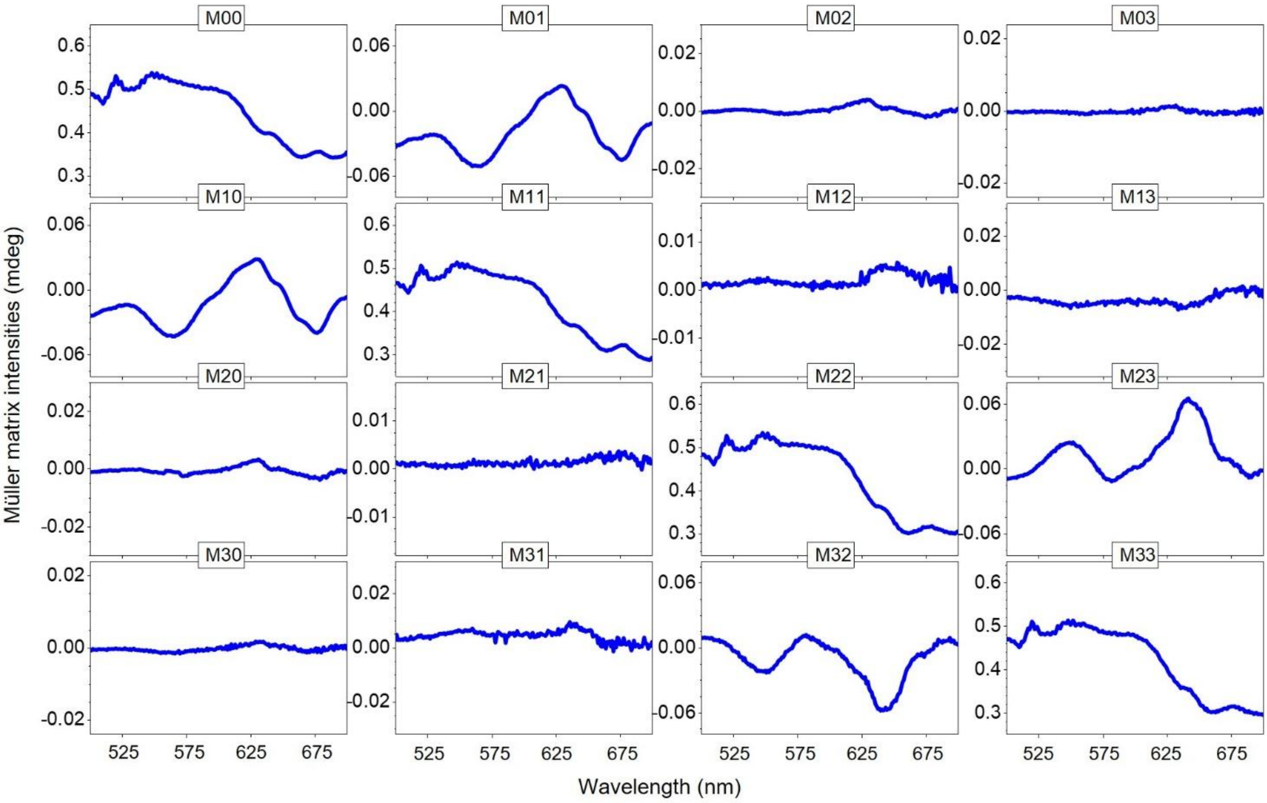

Supplementary Fig. 10 Müller matrix for RS with anti-streptavidin.

All the MMP elements for the RS metamaterial in the buffer after the mobilisation of anti-streptavidin on metamaterial.

12. Müller matrix for LH and RH structure in Buffer

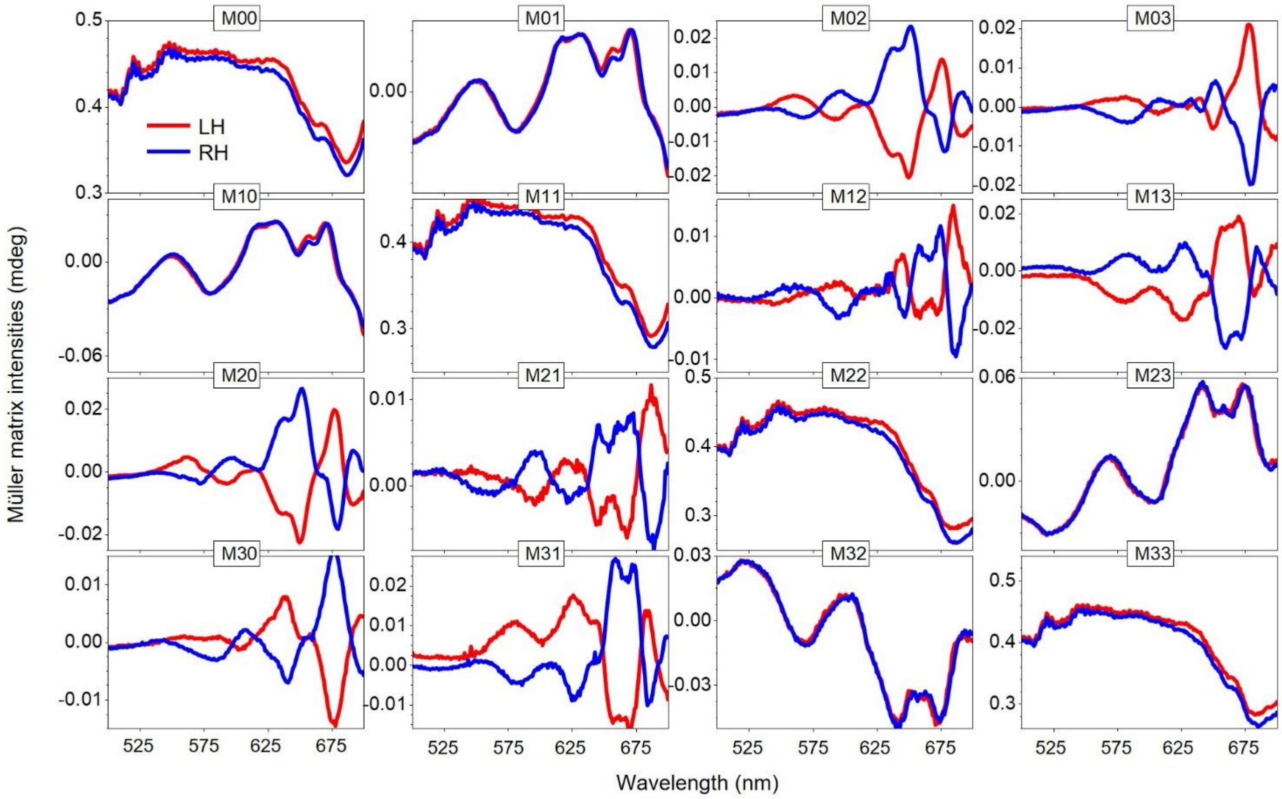

Supplementary Fig. 11 Müller matrix for LH (red) and RH (blue) structures in buffer.

All the MMP elements for the RH metamaterial in the buffer before the mobilisation of streptavidin on metamaterial.

13. Müller matrix for LH and RH structure with Streptavidin

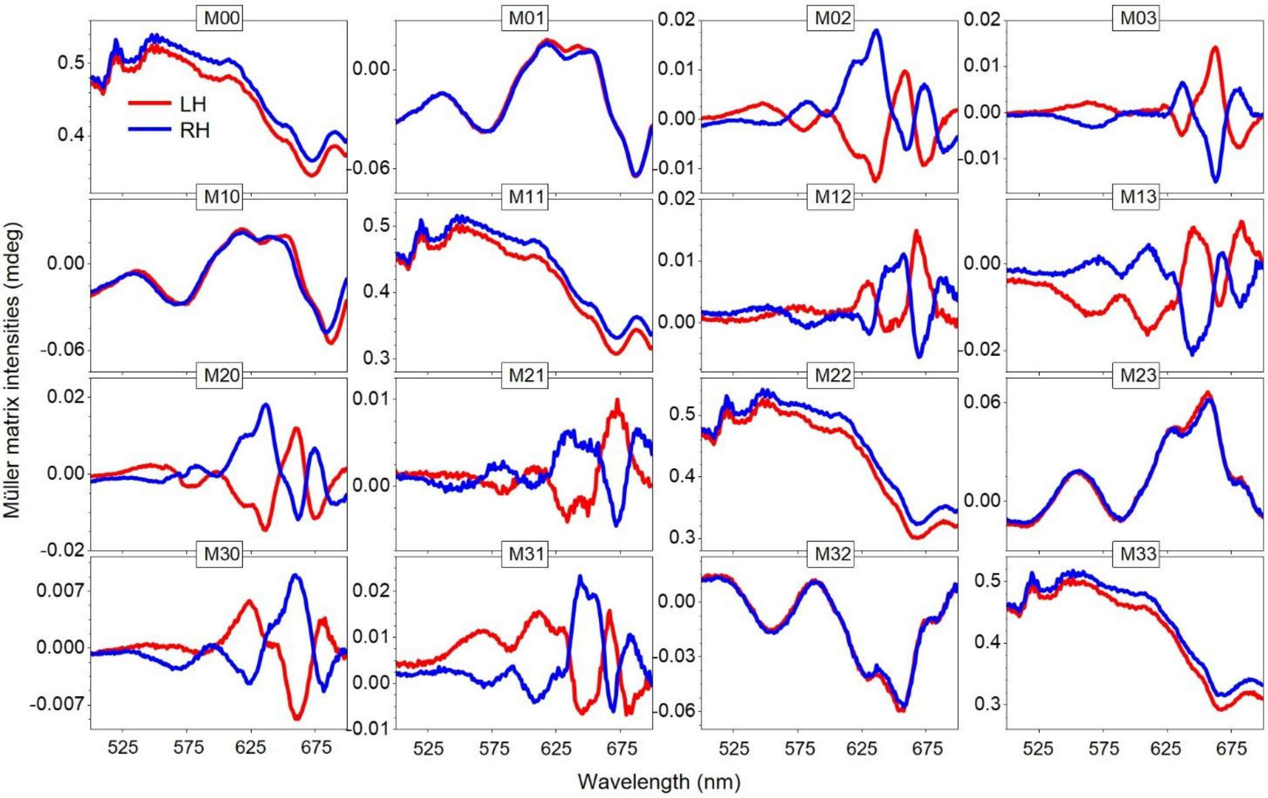

Supplementary Fig. 12 Müller matrix for LH (red) and RH (blue) structures in streptavidin.

All the MMP elements for the LH and RH metamaterial in the buffer after the mobilisation of streptavidin on metamaterial.

14. Müller matrix for LH and RH structure with anti-streptavidin

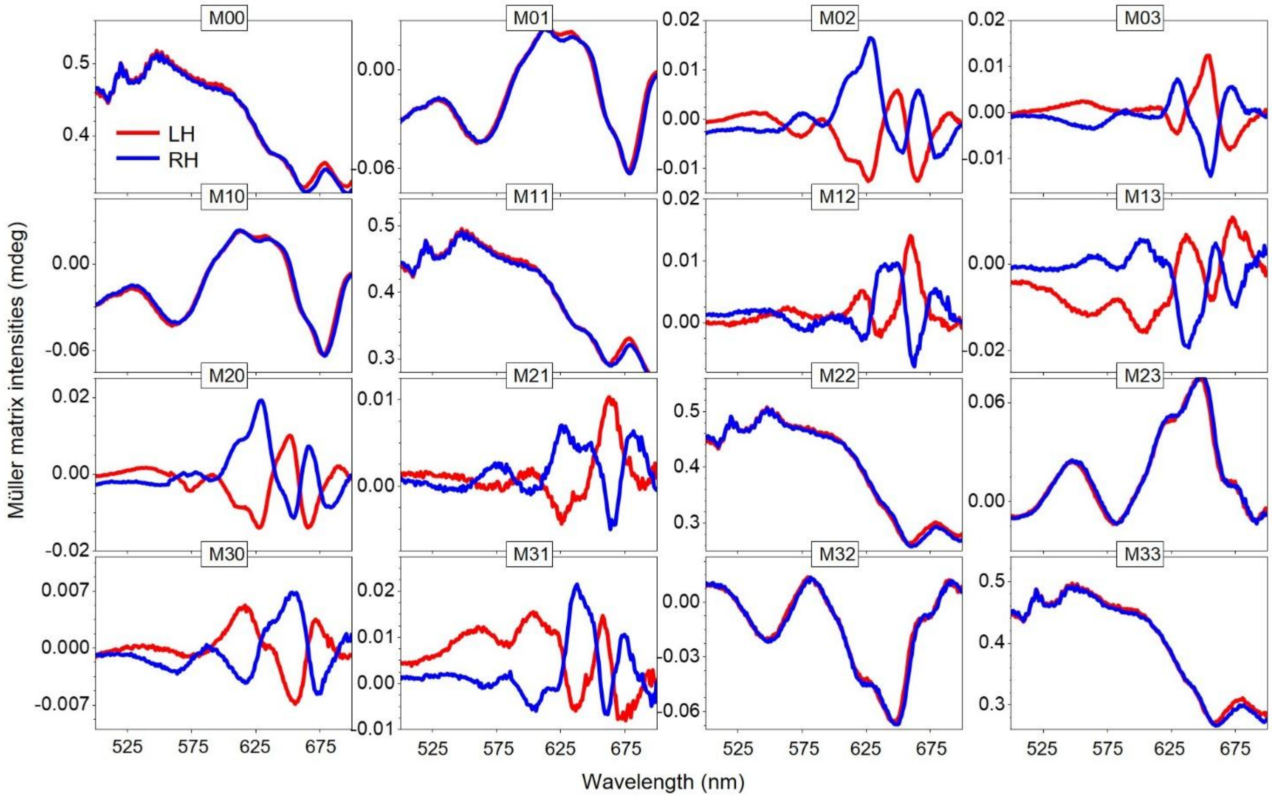

Supplementary Fig. 13 Müller matrix for LH (red) and RH (blue) structures in anti-streptavidin.

All the MMP elements for the LH and RH metamaterial in the buffer after the mobilisation of anti-streptavidin on metamaterial.

15. Reflectivity for LH and RH structure with and without polarisation layer

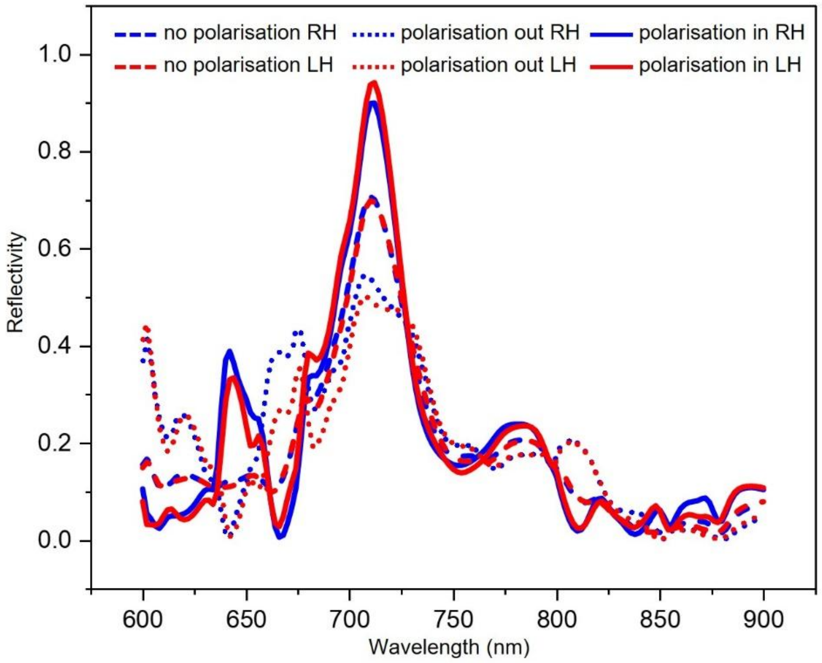

Supplementary Fig. 14 Reflectivity calculated from the numerical simulation.

Calculated reflectivity for LH (red) and RH (blue) metamaterials without polarisation (dashed line), Outward polarisation (solid line) and Inward into metamaterial (dotted line).

16. Magnetic field showing for opposite polarization layer

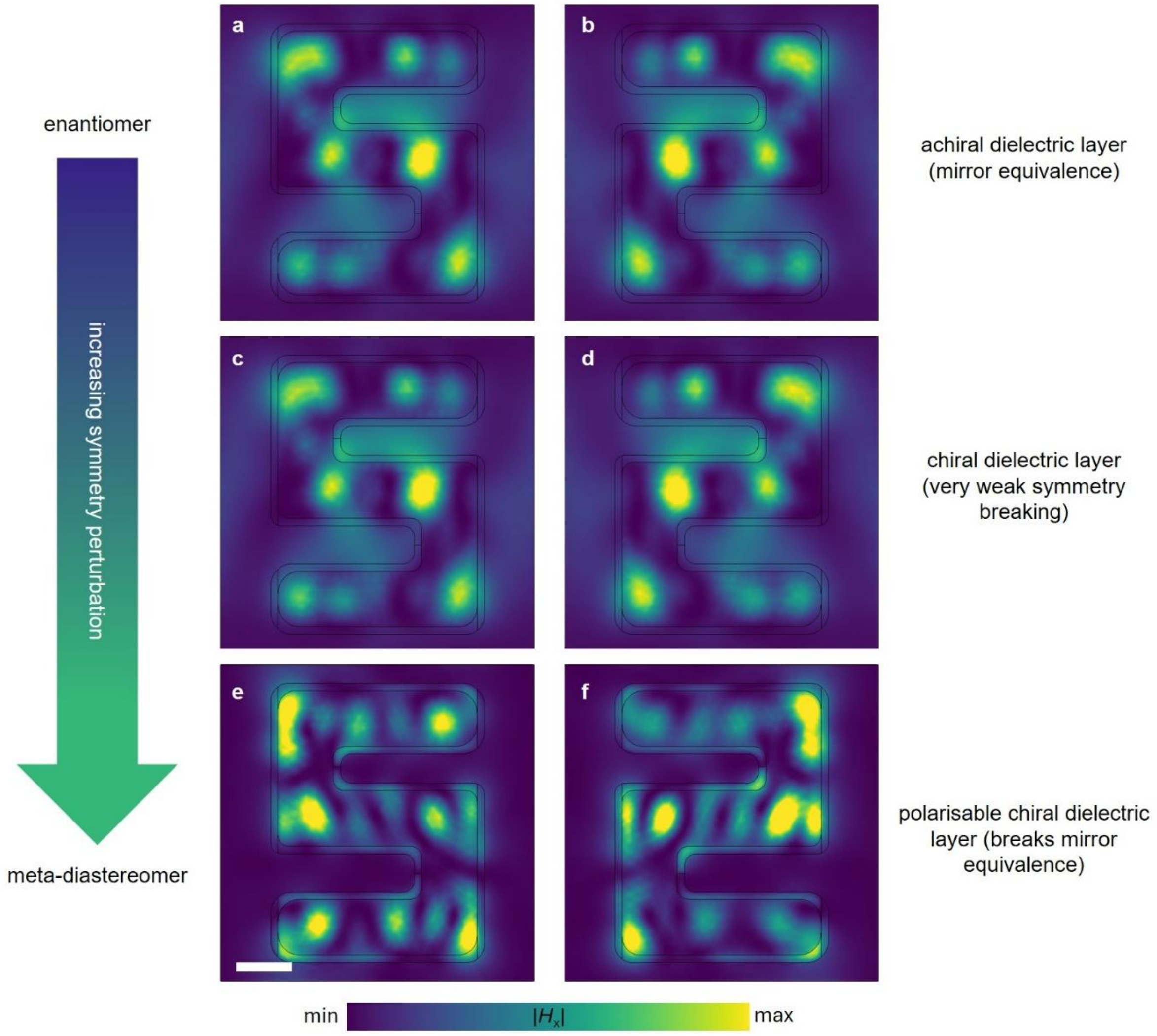

Supplementary Fig. 15 Calculated magnetic field |H_x| for inward polarisation.

Showing the comparison of magnetic field profile |H_x|, at a wavelength of 650 nm. **a,b** without chiral layer; **c,d** with chiral layer but no polarisation; **e,f** with both chiral layer and polarisation layer with polarisation facing inward into the metamaterial. The scale bar is 150 nm.

17. Magnetic field showing for opposite polarization layer showing top surface and slice from middle of S structures

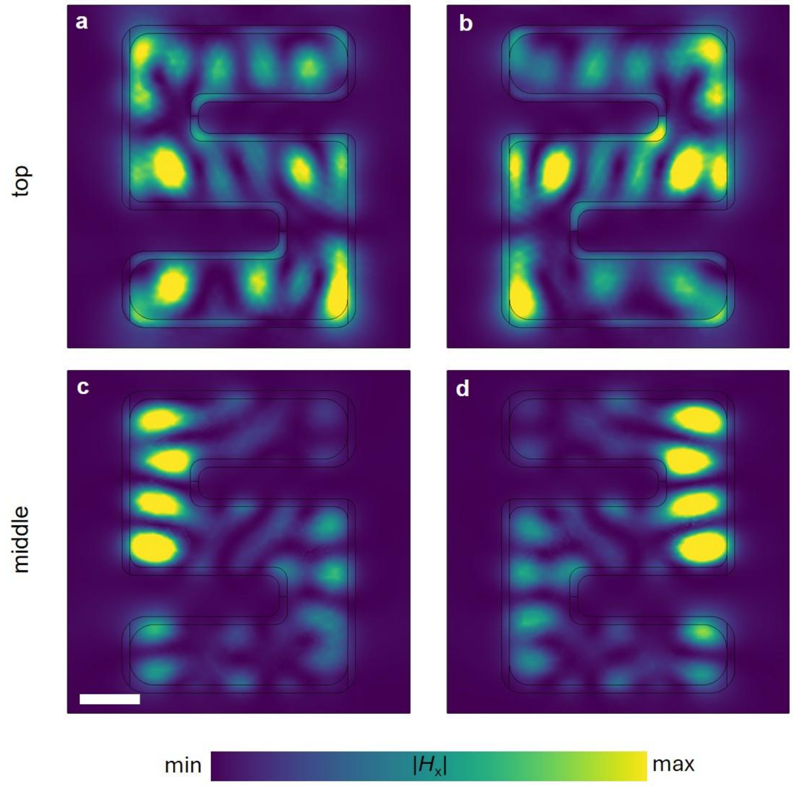

Supplementary Fig. 16 magnetic field profile at interface and inside the metamaterial.

Showing the comparison of magnetic field profile $|H_x|$, at a wavelength of 650 nm. **a,b** The top plane passing through the metamaterial-protein interface of the protein. **c,d** Middle plane passing through the centre of the metamaterial. The scale bar is 150 nm.

18. S_{RH} reflectance spectra

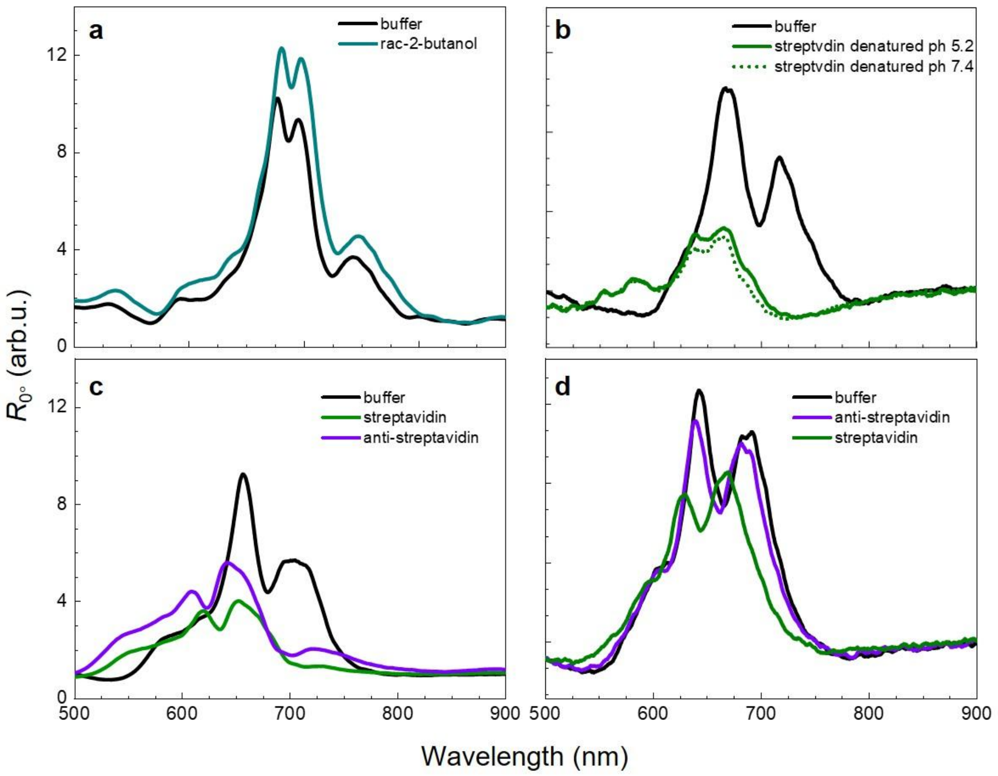

Supplementary Fig. 17 Reflectance response of S_{RH} metasurfaces under dielectric and biomolecular modification.

Reflectance spectra of representative S_{RH} silicon metasurfaces, with the silicon thickness of each sample given in parentheses. Spectra collected with the metasurfaces immersed PBS buffer are shown as solid lines. These are compared with: **a** (210 nm) immersion in rac-2-butanol (dark cyan); **b** (190 nm) immobilised streptavidin (green) followed by binding of anti-streptavidin (purple); **c** (180 nm) immobilised denatured streptavidin measured at pH 5.5 (green) and after exchange back to pH 7.4 buffer (dotted green); and **d** (170 nm) immobilised anti-streptavidin (purple) followed by subsequent binding of streptavidin (green). All spectra were acquired in buffer after each functionalisation or binding step.

19. Jones–Müller interpretation of adsorption-induced linear dichroism

Supplementary Note 1

To complement the boundary-condition formulation presented in the main text, we provide here a Jones–Müller analysis of the adsorption-induced polarimetric response. This formulation clarifies, at the level of symmetry and scattering matrices, why the strongest handedness-dependent adsorption signature appears in linear dichroism (LD), independent of the microscopic parametrisation of the effective surface response[1].

1. Polarisation conventions

We consider normally incident plane waves and define linear basis vectors (x, y) and circular basis vectors (L, R) as

$$\mid L\rangle = \frac{1}{\sqrt{2}}(x + \mathrm{i}y), \mid R\rangle = \frac{1}{\sqrt{2}}(x - \mathrm{i}y). \qquad (1)$$

The metasurface–interface system is described by an effective Jones matrix $\mathbf{J}$ relating incident and transmitted (or reflected) field amplitudes,

$$\mathbf{E}^{\mathrm{out}} = \mathbf{J}\mathbf{E}^{\mathrm{in}}. \qquad (2)$$

In the circular basis, the Jones matrix is written as

$$\mathbf{J} = \begin{pmatrix} J_{\mathrm{LL}} & J_{\mathrm{LR}} \\ J_{\mathrm{RL}} & J_{\mathrm{RR}} \end{pmatrix}. \qquad (3)$$

Here J_{ij} are complex field-amplitude coefficients describing the full resonant metasurface–interface system.

Müller matrix elements may be expressed in terms of Jones elements using standard relations. In the Stokes convention adopted here,

$$m_{30} = \frac{\mid J_{\mathrm{LL}} \mid^2 - \mid J_{\mathrm{LR}} \mid^2 + \mid J_{\mathrm{RL}} \mid^2 - \mid J_{\mathrm{RR}} \mid^2}{2}, \qquad (4)$$
$$m_{10} = \mathrm{Re}\,(J_{\mathrm{LL}} J_{\mathrm{LR}}^* + J_{\mathrm{RR}} J_{\mathrm{RL}}^*), \qquad (5)$$
$$m_{20} = \mathrm{Im}\,(J_{\mathrm{LL}} J_{\mathrm{LR}}^* - J_{\mathrm{RR}} J_{\mathrm{RL}}^*). \qquad (6)$$

Here m_{30} corresponds to circular dichroism (CD), while m_{10} and m_{20} correspond to LD′ and LD′′, respectively (up to overall normalisation factors that do not affect the linear dependence derived below).

2. Boundary-condition model in tensor form

In the main text, adsorption is described through the effective surface current

$$\mathbf{J}_s = \sigma_0(\omega)\mathbf{E}_\parallel + \sigma_\chi(\omega)(\hat{\mathbf{n}} \times \mathbf{E}_\parallel), \qquad (7)$$

which may be written equivalently as an in-plane tensor response

$$\mathbf{J}_s = \mathbf{\Sigma}\mathbf{E}_\parallel, \mathbf{\Sigma} = \sigma_0\mathbf{I} + \sigma_\chi\mathbf{R}_{90}, \qquad (8)$$

with

$$\mathbf{R}_{90} = \begin{pmatrix} 0 & -1 \\ 1 & 0 \end{pmatrix}. \qquad (9)$$

Such tensorial surface descriptions are standard within generalized sheet-transition-condition (GSTC) metasurface electrodynamics[2-5].

Under transformation to the circular basis defined above, the rotation matrix becomes diagonal,

$$\mathbf{R}_{90} \to \begin{pmatrix} +\mathrm{i} & 0 \\ 0 & -\mathrm{i} \end{pmatrix}, \qquad (10)$$

so that

$$\mathbf{R}_{90} \mid L\rangle = +\mathrm{i} \mid L\rangle, \mathbf{R}_{90} \mid R\rangle = -\mathrm{i} \mid R\rangle. \qquad (11)$$

Thus, the antisymmetric chiral surface term in Eq. (7) corresponds to opposite-sign diagonal perturbations for left- and right-handed helicities.

3. Adsorption-induced perturbation of the effective Jones matrix

For the anisotropic S-shaped metasurface, structural anisotropy generally yields non-zero off-diagonal elements J_{LR} and J_{RL}.

Adsorption of a chiral molecular layer introduces a leading-order helicity-dependent perturbation to the diagonal circular elements,

$$J_{\mathrm{LL}} \to J_{\mathrm{LL}} + \varepsilon, J_{\mathrm{RR}} \to J_{\mathrm{RR}} - \varepsilon, \qquad (12)$$

where $\varepsilon(\omega)$ is a small complex quantity representing the adsorption-induced chiral modification of the co-polarised circular scattering amplitude.

To first order, J_{LR} and J_{RL} are taken to remain unchanged, as they arise from intrinsic structural anisotropy of the metasurface. Additional adsorption-induced changes to these elements would generate higher-order corrections without altering the linear coupling derived below.

Within the boundary-condition framework, $\varepsilon(\omega)$ is proportional to the modal overlap of the chiral surface term $\sigma_{\chi}(\omega)$ with the resonant near fields; the symmetry argument below does not depend on the microscopic origin of this perturbation[2-5].

4. Linear sensitivity of LD

Substituting Eq. (12) into Eq. (5) and retaining terms linear in ε yields

$$\Delta m_{10} = \mathrm{Re}\,[(J_{\mathrm{LR}} - J_{\mathrm{RL}})^{*}\varepsilon]. \qquad (13)$$

Equation (13) shows that the adsorption-induced change in LD scales linearly with the chiral perturbation ε and is proportional to the intrinsic structural anisotropy encoded in $J_{\mathrm{LR}} - J_{\mathrm{RL}}$.

By contrast, the corresponding change in circular dichroism, obtained from Eq. (S2), contains dominant magnitude-squared contributions and need not provide an equally clean linear enantiomer-differential signature under mirror inversion.

5. Implications

Equation (13) demonstrates that the strongest handedness-dependent adsorption signature in LD arises naturally from combining:

- intrinsic metasurface anisotropy (non-zero $J_{\mathrm{LR}} - J_{\mathrm{RL}}$), and
- a chiral diagonal perturbation ($\pm\varepsilon$) induced by adsorption.

This result is independent of the specific microscopic parametrisation of the interfacial layer and therefore complements the boundary-condition description presented in the main text[2-5].

Supplementary references

1 Azzam, R. M. A., Bashara, N. M. & Ballard, S. S. Ellipsometry and polarized light. (1977).

2 Glybovski, S. B., Tretyakov, S. A., Belov, P. A., Kivshar, Y. S. & Simovski, C. R. Metasurfaces: From microwaves to visible. *Phys. Rep.* **634**, 1–72 (2016).

3 Holloway, C. L. & Kuester, E. F. Generalized Sheet Transition Conditions for a Metascreen—A Fishnet Metasurface. *IEEE Trans. Antennas Propag.* **66**, 2414–2427 (2018).

4 Asadchy, V. S., Díaz-Rubio, A. & Tretyakov, S. A. Bianisotropic metasurfaces: physics and applications. *Nanophotonics* **7**, 1069–1094 (2018).

5 Achouri, K. & Caloz, C. Design, concepts, and applications of electromagnetic metasurfaces. *Nanophotonics* **7**, 1095–1116 (2018).
