# Supplementary material for: Metasurface analogues of molecular diastereomers from hierarchical multiscale chiral interactions with biomolecules
Source: Nat Commun. 2026 Apr 22;17:5541. doi: 10.1038/s41467-026-72200-6 (PMC13287669; doi:10.1038/s41467-026-72200-6)
Supplement: Supplementary file 2 — Description of Additional Supplementary Files [file 41467_2026_72200_MOESM2_ESM.pdf]

Description of Additional Supplementary Files

File Name: Supplementary Data 1

Description: Unprocessed Raw Data
