# Supplementary material for: Metasurface analogues of molecular diastereomers from hierarchical multiscale chiral interactions with biomolecules
Source: Nat Commun. 2026 Apr 22;17:5541. doi: 10.1038/s41467-026-72200-6 (PMC13287669; doi:10.1038/s41467-026-72200-6)
Supplement: Supplementary file 5 — Transparent Peer Review file [file 41467_2026_72200_MOESM5_ESM.pdf]

nature portfolio

Peer Review File

Metasurface analogues of molecular diastereomers from hierarchical multiscale chiral interactions with biomolecules

Corresponding Author: Professor Malcolm Kadodwala

This file contains all reviewer reports in order by version, followed by all author rebuttals in order by version.

Version 0:

Reviewer comments:

Reviewer #1

(Remarks to the Author)
This manuscript presents a concept of “meta-diastereomers” which combines molecular chirality with nanoscale enantiomeric structures to explore hierarchical chiral interactions and their optical properties. The application of meta-diastereomers in probing antibody-antigen interactions is also carried out to highlight their potential for label-free biosensing and diagnostics. The innovation of this work deserves further consideration by Nature Communications but the current manuscript needs to be fully polished. The following issues are provided for the authors.
1、The Abstract part of the current manuscript is somewhat simplistic. For instance, there should be previous reports concerning the chiroptical activities integrating chirality across different scales. The authors are encouraged to enrich this section by incorporating relevant contextual introductions and highlighting the unique advancement of this work.
2、A more precise definition for the “meta-diastereomers” is necessary for the revised manuscript, including a comparison with traditional diastereomers and chiral systems. Can the authors give some illustrative examples which would be beneficial for better understanding by readers. Additionally, the authors should discuss how meta-diastereomers relate to or differ from other hierarchical chiral systems reported in the previous literature such as molecular diastereomers.
3、The experimental sections particularly the fabrication processes and characterizations can be elaborated for easy reproducibility. Specific parameters (e.g., concentrations and incubation times) should be clearly stated.
4、Similar issues are also applicable to simulation modeling. For instance, how the choice of simulation parameters impacts the blue shift of Abs. should be elaborated.
5、Additionally, the authors are strongly suggested to improve the image resolution in the Supplementary Information. For example, Figure S2 in the Supplementary Information is rather poor in resolution and one cannot catch any important information.
6、LD and pure CD signs normally concurrently appeared during chiroptical characterizations. How to exactly characterize them separately in Figure 3.
7、Racemic 2-butanol was adopted to test the optical response of resultant meta-diastereomers to the refractive index (RI) change of the surrounding environment. However, possible chirality-dependent enrichment of racemic 2-butanol would influence the conclusion. Hence, achiral solvent is suggested to be additionally tested.
8、The detection performances should be compared with results reported by other methods or systems in order to highlight advantages and limitations.
9、The format of some references in the current manuscript is not unified, such as the number of authors, the name of the document, the DOI number, etc.

Reviewer #2

(Remarks to the Author)
The authors have introduced a novel metasurface based on silicon nanostructures and demonstrated its optical properties using various spectroscopic techniques (e.g., linear dichroism, circular dichroism). They also attempted to theoretically explain the experimentally obtained spectra through numerical simulations. Additionally, the authors measured and explained the optical changes induced by treating the metasurface with a chiral antigen (streptavidin). Furthermore, they investigated the optical changes that occurred after treating the metasurface with an antibody capable of binding to the antigen, showcasing the potential applicability of this metasurface in biosensing and other fields.
While this manuscript presents a new avenue for hybrid systems with multi-scale chirality, it lacks experimental analyses

beyond spectroscopy for characterizing the metasurface, provides insufficient explanations for experimental and numerical simulation results, and unfortunately does not experimentally demonstrate applications of this promising technology. Therefore, I do not recommend that this manuscript not be accepted for publication in Nature Communications. Below are other comments for the manuscript, hopefully to improve its quality:
1. The authors attributed the difference in spacing between the top, middle, and bottom arms of the S-shaped structure to the C1 point group in lines 66–68 of the manuscript. However, it is unclear whether the C1 point group refers to the silicon atom or the S-shaped structure. Additionally, it raises the question of whether the authors intentionally designed the metasurface with varying spacing between the arms during the fabrication process. The authors mentioned using e-beam lithography for the metasurface fabrication, and considering the resolution of e-beam lithography, it seems plausible that, if the authors intended to maintain uniform spacing between the arms, it could have been achieved.
2. The authors conducted reflectance measurements and numerical simulations of unfunctionalized metasurfaces using rac-2-butanol as the solvent in addition to PBS buffer. While the use of PBS buffer is understandable given its necessity in the measurement process of functionalized metasurfaces, it is unclear why rac-2-butanol was specifically chosen for reflectance measurements and numerical simulations. An explanation for this choice seems necessary.
3. The authors presented the CD spectra of racemic metasurfaces in Figures S3e and S3f. However, since both RA and RS metasurfaces exhibit significant non-zero CD signals, it raises doubts about whether they are truly racemic. Specifically, the CD spectra of the racemic metasurfaces provided by the authors closely resemble those of the RH metasurface. I believe additional explanation on this matter is necessary.
4. The authors assumed a dipole layer composed of streptavidin surrounding the S-shaped structure to analyze the cause of the blue-shift observed in various spectra when streptavidin is attached to the metasurface. As shown in Figure 6c, they assumed that the polarization direction of the dipole layer consistently points inward toward the S-shaped structure. Meanwhile, when the authors attached streptavidin to the surface of the S-shaped structure, they used glutaraldehyde to link polylysine and streptavidin. However, since glutaraldehyde induces crosslinking between the abundant amine groups within proteins rather than targeting specific protein or peptide sequences, it raises doubts as to whether streptavidin on the metasurface surface could uniformly attach in a specific direction as depicted in Figure 1c. Therefore, to proceed with simulations assuming, as the authors suggested, that the polarization direction of the dipole layer consistently points inward toward the S-shaped structure, the dipole moment of the streptavidin-treated metasurface should be experimentally demonstrated.
5. The authors presented the asymmetry parameter based on the magnitude of peak R on page 6 and in Table 1. However, as the authors themselves mentioned, the asymmetry parameter for αCDBuffer should theoretically be 1. Even accounting for experimental error, a value of 1.37 appears to deviate significantly from the theoretical value of 1. The authors attributed this discrepancy to "the non-chirally sensitive contribution to the CD due to the depolarising properties of the S structures." However, a more detailed explanation of this claim seems necessary.
6. The authors used a polarization value of 1×10−8 Cm−2 for streptavidin in their numerical simulations of the functionalized metasurface. However, this value differs significantly from the value of 8×10−4 Cm−2 cited in the referenced literature. The authors stated that they used 1×10−8 Cm−2 to achieve "qualitative agreement with the experiment," but they did not provide sufficient justification or references to support this choice. The authors need to explain the rationale for conducting numerical simulations using a polarization value that differs by a factor of 8×104 from previous research findings.
7. The authors stated that this metasurface could be applied to biosensing and other areas. To support this claim, it would be more convincing if they experimentally demonstrated the ability of this metasurface to function as a biosensor. For example, by slightly modifying the experimental methods presented in the manuscript, the authors could immobilize anti-streptavidin antibodies on the surface of the metasurface and treat it with streptavidin. The resulting spectral changes could then illustrate its potential as a streptavidin biosensor.
8. The authors demonstrated that the asymmetry parameter decreased when anti-streptavidin antibodies were treated in addition to streptavidin on the metasurface. To explain this, they stated that the binding of anti-streptavidin caused a decrease in the anisotropy of the alignment of the dipole moment, as shown in Figure 8. However, as the authors themselves pointed out, while the asymmetry parameter decreased, the blue-shift in the spectra actually increased. The authors should provide a more detailed explanation for the decrease in the asymmetry parameter and the simultaneous increase in the blue-shift observed upon the addition of anti-streptavidin.

Reviewer #3

(Remarks to the Author)
This work is about the influence of adsorption of chiral biomolecules to asymmetric S-shaped silicon nanostructure arrays on the (chir)optical response of the arrays. The authors claim that they form meta-diastereomers through the interaction of the chiral biomolecules with the two different silicon S-shaped enantiomers, in the sense that they have coupling of the (chirotical?) properties between the two objects that are nearly two orders of magnitude differrent in size.
I think that the work is interesting and novel. While there has been a lot of work on chirality and Mie resonances in dielectric nanostructures, I did not see something like this concept of meta-diastereomers. The question is whether this concept is valid and significant, and what would be the difference between this concept, and just having the dipolar or multipolar electric/magnetic resonances of the dielectric features interacting with chiral molecules at the surface of the structure, as described in seevral publications of Dionne and coworkers, for example. I think that the authors should explain clearly how is their proposed concept different, if it is different?
There is one experimental finding, of a blue-shift of the main magnetic resonance of the S-shape with adsorption of streptavidin, which cannot be explained by a change in the local refractive index. The authors claim that it is a consequence of the charged surface formed by adsorption of the protein. They perform a simple model simulation, where instead of describing the charged surface, they attribute a polarization layer to the surface of the nanostructures, which seems to produce the same effect.

Another unique feature, is that the addition of another protein, anti-strep, seems to null the effect. The authors try to explain this by a change in the charging state of the surface.
I find the paper not convincing enough and not so clear for the general reader:
1. I think that the non-expert reader will find it hard to understand what is unique here vs. other models of interaction of Mie resonances with chiral molecules, as I wrote above. I suggest to explain this in simple terms.
2. I would feel much more comfortable recommending the paper for publication, if the authors could demonstrate the blue shift for simpler cases where charging could be better controlled and perhaps also switch the blue shift on/off. What would happen if the pH of solution around adsorbed polylysine would be changed to neutralize it? Or some other polyelectrolyte?
3. The panels of the experimental results in Fig. S2 are difficult to read. Perhaps better to bunch together the individual component spectra, like the CD (03) before and after the various adsorption steps, and display them in a larger, clearer format. Part of the data appears in Fig. 3, but one could do it also with the rest of the data in Fig. S2. Also in Fig. 3, it could be helpful to also show spectra of all three cases (for a single handedness) in one panel for easier comparison between the features of the spectra, as the changes are not so large and the sepctra contain many peaks.
4. Page 4, line 96, should be “(Figure 2a)” not “1a”

After clarifications about the uniqueness of these meta-stereomers and perhaps more compelling experimental data about the nature of the blue-shift and anti-strep effects, the paper could become suitable for publication in Nat. Comm.

Version 1:

Reviewer comments:

Reviewer #1

(Remarks to the Author)
The authors have made substantial structural and conceptual revisions to the manuscript. The introduction of a rigorous electromagnetic boundary-condition formulation successfully provides a quantitative basis for interpreting the observed spectral behavior. The authors have thoroughly addressed my previous concerns and incorporated valuable new experimental data, including conformational controls and measurements across the isoelectric point. These additions significantly elevate the scientific rigor and logical consistency of the work. However, a minor inconsistency persists in Figure 4. In the caption for Figure 4, the text states, “The R parameter is defined in the supplementary information...”. The accompanying table in Figure 4d defines the asymmetry parameter using the symbol α (e.g.,), and the main text refers to “peak R” as the resonance associated with the magnetic-dipole mode. The authors should correct the caption to refer to the “α parameter” or “asymmetry parameter” rather than the “R parameter” to avoid confusing the parameter itself with the label for the resonance peak.

Overall, I would like to recommend acceptance of the manuscript after the minor typographical issue noted above is addressed.

Reviewer #2

(Remarks to the Author)
The revised manuscript demonstrates a substantial improvement in both scholarly completeness and overall contribution relative to the original submission. As detailed in the authors’ Response to Referees, the manuscript has been extensively revised at both the structural and conceptual levels. Notably, the concept of the “meta-diastereomer,” which was a shared concern among the reviewers, is now presented with much greater clarity. The experimental design and interpretation of the results have also been significantly strengthened, and the newly added computational simulations are in much closer agreement with the experimental findings. Consequently, the main contribution of the work has been successfully repositioned from the originally proposed biosensing platform toward the identification of a boundary-mediated mechanism through which molecular chirality is translated into the optical response. In this respect, the manuscript is now much improved and, in my view, is potentially suitable for publication after minor revision. The remaining issues are primarily related to clarity of presentation and consistency of notation.

1. On Page 5, Line 2, the authors state that even when the same fabrication process is employed, the silicon thickness varies slightly within the range of 170–210 nm. This variation is entirely understandable, and the authors have appropriately specified the silicon thickness used in each experiment when describing the results in Fig. 3, Fig. 5, and related sections, which improves the rigor of the manuscript. However, in the current presentation, readers may still be left uncertain as to whether the differences in silicon thickness reported across experiments simply reflect unavoidable sample-to-sample variation arising from the fabrication process, or whether silicon thickness was intentionally controlled as an experimental variable. It would therefore be helpful if the descriptions of the silicon thickness used in the experiments were revised throughout the manuscript in a more explicit and reader-friendly manner.
2. On Page 5, Line 11, the manuscript states: “Absorbance (Abs) and depolarization Index (0≥DI≤1) …”. This appears to be a typographical error and presumably should read “(0≤DI≤1)”. Likewise, on Page 5, Line 13, “0.5≥DI≤1” also appears to be a typographical error and likely should read “0.5≤DI≤1”. If so, these expressions should be corrected.
3. In Fig. 3, the inclusion of an appropriate legend would improve readability. At present, the measurement conditions corresponding to each graph are described only in the figure caption, which makes the figure somewhat less intuitive to interpret. In addition, all of the graphs are presented in the same color (red), which further reduces visual clarity. I therefore

suggest using clearly distinguishable colors for the different graphs and including a corresponding legend directly within the figure.
4. In Fig. 4a–c, the x-axis ticks should be indicated more clearly. Currently, only Fig. 4c provides numerical labels for the x-axis range and tick positions, and even there, some graphs appear to show slight inconsistencies between the grid and tick positions. I therefore recommend that the x-axis ticks in each of Fig. 4a–c be presented more clearly and accurately.
5. On Page 10, Line 4, “fig. 4d” is written in lowercase. For consistency with the notation used throughout the manuscript, I suggest revising this to “Fig. 4d”.

Reviewer #3

(Remarks to the Author)
I read the response of the authors to the reviewers' comments and the revised manuscript and I find the paper significantly improved in the clarity of the proposed model and physical decription of it.
I support publication, since I do not have a better explanation for the blue shift, although I am still not totally convinced about the boudary layer model. I would have still loved to see some more experimental proff for it. For example, considering eq. 3, I would assume that if the protein at the interface would have been replaced with a metal nanoparticle film, wouldn't the high polarizability of the metal increase the polarization effect significantly? in that case wouldn't the blue shift become more substantial? Or maybe it will scramble the whole reflectance spectrum completely, and then it will not be so useful? Just a thought to consider in order to strengthen the proposed model.
Another point: A possible explanation about the difference in optical responses between the chiral liquid and the proteins could be due to aligned adsorption vs. total randomness in chiral butanol molecules orientation. I know that the authors claim that the glutaraldehyde process does not leave the protein aligned with respect to the surface, but I am not sure if this is correct.
Figure 3b: the dotted line for the adsorbed strep cannot be seen in the graph.

Open Access This Peer Review File is licensed under a Creative Commons Attribution 4.0 International License, which permits use, sharing, adaptation, distribution and reproduction in any medium or format, as long as you give appropriate credit to the original author(s) and the source, provide a link to the Creative Commons license, and indicate if changes were made.
In cases where reviewers are anonymous, credit should be given to 'Anonymous Referee' and the source.
The images or other third party material in this Peer Review File are included in the article’s Creative Commons license, unless indicated otherwise in a credit line to the material. If material is not included in the article’s Creative Commons license and your intended use is not permitted by statutory regulation or exceeds the permitted use, you will need to obtain permission directly from the copyright holder.
To view a copy of this license, visit https://creativecommons.org/licenses/by/4.0/

We thank the reviewers for their insightful evaluation and constructive suggestions. In response, the manuscript has been substantially revised both structurally and conceptually. The meta-diastereomer framework is now placed within a rigorous electromagnetic boundary-condition formulation, which provides a quantitative basis for interpreting the observed spectral behaviour. Within this framework, adsorption of a charged, polarisable biomolecular interfacial layer modifies the electromagnetic boundary conditions of the resonator, breaking mirror equivalence between the enantiomorphic structures.

To support this formulation, we have incorporated new experimental data, including reciprocal streptavidin/anti-streptavidin binding studies, conformational (denatured) controls, and measurements across the isoelectric point. These additions allow us to distinguish the observed chiroptical response from conventional refractive-index loading or simple surface charge effects.

Response to Reviewer 1

Reviewer 1 Comment 1

"The concept of 'meta-diastereomers' is interesting but feels insufficiently substantiated. The physical origin of the observed signals remains unclear, and the study feels somewhat preliminary for a journal of this caliber."

Response:

We thank the reviewer for this assessment. We agree that the original submission did not sufficiently articulate the physical origin of the observed signals. The manuscript has now been revised to clarify the boundary-condition basis of the effect and to provide additional experimental and numerical support.

We now provide a formal physical definition of meta-diastereomers. They are defined as symmetry-inequivalent hybrid states that arise when a charged, polarisable chiral interfacial layer perturbs the electromagnetic boundary conditions of an enantiomorphic dielectric resonator. This formulation places the concept explicitly within boundary-value electrodynamics.

The revised manuscript introduces a chiral extension of established electromagnetic boundary-condition theory. Classical treatments show that charged interfacial layers can induce resonance shifts in achiral systems through boundary modification. We demonstrate that when the interfacial layer is chiral and the resonator lacks mirror symmetry, the boundary perturbation acquires handedness-dependent contributions. These contributions break mirror equivalence and render linear optical observables stereostructurally informative.

A symmetry-resolved Jones–Mueller analysis is introduced to identify the conditions under which linear dichroism (LD) becomes sensitive to molecular stereostructure. This formalism expresses the boundary-mediated symmetry breaking and clarifies why the effect appears in linear channels.

Additional experimental controls and modelling have been incorporated. Measurements across the isoelectric point of streptavidin (new Fig. 3) demonstrate that the blue shift persists irrespective of the sign of the net protein charge. Comparison of native and denatured protein layers (new Fig. 5) establishes sensitivity to conformational state. Expanded numerical modelling (new Fig. 7; SI S3, S7) shows that effective-medium refractive-index loading cannot reproduce the observed behaviour, whereas boundary-condition perturbation accounts for both the direction and magnitude of the spectral shifts.

These additions clarify the physical origin of the signals and address the concern regarding insufficient substantiation.

Reviewer 1 Comment 2

"The Abstract is somewhat simplistic and should better contextualize prior work integrating chirality across scales."

Response:

The Abstract has been rewritten to situate the work within prior efforts to integrate molecular and nanoscale chirality. It now distinguishes the present boundary-mediated mechanism from superchiral near-field enhancement strategies and bulk constitutive chiral descriptions, and explicitly states the conceptual contribution.

Reviewer 1 Comment 3

"A more precise definition for 'meta-diastereomers' is necessary, including comparison with traditional diastereomers and hierarchical chiral systems."

Response:

The Introduction now provides a precise definition of meta-diastereomers and explicitly compares them with molecular diastereomers. In both cases, symmetry-inequivalent hybrid states arise whose measurable properties depend on the relative handedness of coupled components. We distinguish this framework from hierarchical chiral systems in which chirality is treated as scale-specific rather than as an interfacial boundary-mediated phenomenon.

Reviewer 1 Comment 4

"The experimental sections particularly the fabrication processes and characterizations can be elaborated for easy reproducibility."

Response:

The Methods section has been expanded to include detailed fabrication parameters, including silicon thickness ranges (170–210 nm), lithographic patterning, protein concentrations, incubation times, buffer conditions, and the full sequence of surface functionalisation (PLL adsorption, glutaraldehyde cross-linking, protein immobilisation, ethanolamine blocking). Additional details are provided in the Supplementary Information.

Reviewer 1 Comment 5

"Simulation modelling should elaborate how parameter choice impacts the blue shift."

Response:

The simulation section has been expanded (new Fig. 7; SI S3, S7). We explicitly compare simulations based on effective refractive-index loading with those incorporating boundary-condition

perturbation. The inability of effective-medium modelling to reproduce the blue shift is demonstrated. Additional detail is provided regarding unit-cell construction for racemic arrays (SI S7) and implementation of boundary conditions.

Reviewer 1 Comment 6

"LD and pure CD normally appear concurrently. How are they separated in Figure 3?"

Response:

Full Mueller matrix polarimetry was used. LD', LD'', CD, and related terms were extracted via analytic inversion of the Mueller matrix. The relevant Mueller matrix elements are shown explicitly (SI S5), and the decomposition procedure is described in the Methods. The role of depolarisation is clarified, and the separation of linear and circular contributions is explained.

Reviewer 1 Comment 7

"Racemic 2-butanol was adopted… possible chirality-dependent enrichment… achiral solvent suggested."

Response:

Racemic 2-butanol was used to benchmark refractive-index sensitivity. As shown in SI S1, reflectance spectra in racemic, (R)- and (S)-2-butanol are indistinguishable for LH and RH metasurfaces, and the magnetic dipole resonance position is insensitive to molecular chirality under dielectric loading alone. This establishes that neutral chiral liquids do not produce handedness-dependent effects in this system. The text has been revised to clarify this explicitly.

Reviewer 1 Comment 8

"Better benchmarking against established chiral biosensing platforms, specifically comparing LOD."

Response:

We appreciate the reviewer's emphasis on analytical benchmarking. In the original submission, the work was framed more strongly in a biosensing context. In the revised manuscript, we clarify that the primary contribution is the identification of a boundary-mediated mechanism by which molecular chirality is transmitted into the optical response.

To provide quantitative context, we explicitly report the spectral shifts observed. Streptavidin immobilisation produces a blue shift of approximately 15 nm. By comparison, refractive-index benchmarking using racemic 2-butanol produces a ~3 nm red shift corresponding to ~48 nm RIU^{-1}. This RI sensitivity is substantially lower than that typically reported for plasmonic metafilms optimised for bulk refractive-index detection.

Importantly, the metasurface shows no handedness-dependent response to neutral chiral liquids (SI S1). In contrast, adsorption of charged chiral biomolecules produces both a larger spectral shift and a pronounced handedness-dependent linear dichroism response.

This contrast indicates that the observed effect is not governed by bulk refractive-index contrast or generic molecular chirality in the surrounding medium. Rather, it arises from handedness-dependent perturbation of the electromagnetic boundary conditions at the interface. The response is therefore controlled by interfacial polarisation and symmetry breaking rather than by analyte mass alone.

For this reason, direct comparison with traditional RI-based LOD values, which assume a linear relationship between surface mass and resonance shift, is not strictly equivalent. The Discussion has been revised to clarify this distinction. The quantitative spectral shifts are reported transparently so that practical sensitivity can be assessed while clarifying the scope of the study.

Reviewer 1 Comment 9

"Reference formatting inconsistent."

Response:

All references have been reformatted for consistency in author listing, journal titles, and DOI presentation.

Final Response to Reviewer 2

Reviewer 2 Comment 1

"The numerical modelling provided does not clearly justify the observed blue shift. Dielectric loading typically leads to a red shift. The authors must prove that their simulation parameters are not arbitrary and that this blue shift is physically grounded."

Response:

We thank the reviewer for this important point. We explicitly address the red-shift versus blue-shift discrepancy by contrasting two distinct physical models in the revised manuscript (new Fig. 7; SI S3, S7).

Conventional dielectric loading.
When the protein layer is modelled as a uniform dielectric shell (n = 1.45), the magnetic dipole resonance consistently red shifts, independent of layer thickness. This confirms that effective-medium or bulk refractive-index loading cannot account for the experimentally observed blue shift.

Boundary-condition perturbation.
We therefore implemented the boundary-condition formalism derived in Eqs. (3) and (4). In this framework, the biomolecular interface is treated as a polarisable interfacial layer that modifies the electromagnetic boundary conditions at the silicon surface. The additional surface response terms perturb the tangential field continuity relations governing the magnetic dipole resonance.

This boundary-mediated model reproduces both the direction (blue shift) and magnitude of the experimentally observed spectral change. The blue shift therefore follows directly from the boundary-condition physics expressed in Eqs. (3) and (4), rather than from bulk dielectric loading. The parameters used are chosen within physically reasonable interfacial response ranges and are not adjusted to force a sign change.

These simulations demonstrate that the observed blue shift is a direct consequence of boundary-condition perturbation and cannot be explained within conventional effective-medium theory.

Reviewer 2 Comment 2

"The specificity of the 'meta-diastereomer' effect to chiral molecules is not fully proven. Could an achiral but charged layer produce similar results?"

Response:

This distinction is clarified in the revised manuscript.

A charged but achiral interfacial layer can modify the electromagnetic boundary conditions and induce a resonance shift through Eqs. (3) and (4). However, such a layer does not reduce mirror symmetry between left- and right-handed resonators and therefore cannot produce a handedness-dependent linear dichroism (LD) response.

The Jones–Mueller symmetry analysis identifies the symmetry conditions under which LD becomes stereostructurally informative. A handedness-dependent response requires the interfacial perturbation itself to break mirror equivalence.

Experimentally, we observe that the blue shift persists across the isoelectric point of streptavidin (Fig. 3), demonstrating that the resonance shift is not determined by the sign of the net charge. However, the handedness-dependent LD response is sensitive to protein conformation and binding state (Fig. 5). This behaviour is consistent with a symmetry-mediated boundary perturbation rather than with a purely electrostatic mass-loading effect.

Reviewer 2 Comment 3

"The manuscript lacks a clear explanation of how the ionic atmosphere around the protein contributes to the optical response."

Response:

The revised manuscript and SI S3 clarify that the interfacial layer is treated as a composite comprising the protein, its hydration shell, and associated counter-ions. This composite layer introduces interfacial polarisability that modifies the electromagnetic boundary conditions of the silicon resonator.

Within the boundary-condition framework of Eqs. (3) and (4), this interfacial layer contributes additional surface response terms that perturb the magnetic dipole resonance via modified tangential field continuity conditions. The resulting spectral shift arises from boundary modification rather than from bulk refractive-index contrast.

When this interfacial layer is chiral and coupled to an enantiomorphic resonator, the perturbation acquires handedness-dependent contributions, producing the observed linear dichroism response.

Reviewer 2 Comment 4

"The choice of silicon thickness and its impact on the magnetic dipole (MD) resonance should be clarified."

Response:

The silicon thickness range (170–210 nm) is now explicitly stated in the Methods. This thickness range positions the magnetic dipole resonance within the spectral window of measurement.

Field distributions shown in SI S7 indicate strong electric and magnetic field localisation at the silicon interface for the magnetic dipole mode. This interfacial localisation makes the MD resonance particularly sensitive to modifications of the electromagnetic boundary conditions, consistent with the mechanism described by Eqs. (3) and (4).

Reviewer 2 Comment 5

"The extraction of Linear Dichroism (LD) from the Mueller Matrix should be better documented to ensure no artifacts from Circular Dichroism (CD) are present."

Response:

Full Mueller matrix polarimetry was performed. The analytical inversion procedure used to extract LD’, LD’’, and CD is detailed in SI S5. The relevant Mueller matrix elements are presented explicitly, and the decomposition separates linear and circular contributions.

The revised Supplementary Information also discusses depolarisation effects and their treatment in the inversion procedure. The extracted LD signals are therefore not artifacts of circular dichroism or depolarising contributions.

Response to Reviewer 3

Reviewer 3 Comment 1

"The reviewer questions whether the proposed meta-diastereomer concept is genuinely distinct from previously reported dipolar or multipolar interactions between dielectric Mie resonances and chiral molecules."

Response:
We have revised the Introduction and Discussion to clarify this distinction. The manuscript now explicitly contrasts the present boundary-mediated framework with conventional near-field enhancement and multipolar coupling models.

In previously reported systems, chiral molecules are typically treated as perturbative dipoles interacting with enhanced local fields of dielectric resonators. In the present work, the optical response arises from adsorption-induced modification of the electromagnetic boundary conditions of the resonator (Eqs. 3–4). The interfacial layer is introduced as a surface-response term that perturbs the tangential field continuity conditions of the silicon structure.

This distinction is supported by additional modelling:

- Simulations treating the adsorbed protein layer as a dielectric shell ($n \approx 1.45$) produce only red shifts and do not reproduce the experimentally observed blue shift (new Fig. 7; SI S3, S7).
- Incorporation of the boundary-condition formulation accounts for both the direction and magnitude of the observed spectral shift.

These revisions clarify how the present framework differs from conventional Mie–molecule coupling models.

Reviewer 3 Comment 2

"The reviewer is not convinced that the blue shift is sufficiently justified and suggests that it may arise from surface charging."

Response:
We have strengthened both the experimental and modelling evidence addressing the origin of the blue shift.

- Simulations treating the adsorbed protein as a dielectric shell ($n \approx 1.45$) produce only red shifts, inconsistent with experiment (new Fig. 7; SI S3).
- Measurements performed above and below the isoelectric point of streptavidin ($pI \approx 5.0$) show that the blue shift persists despite reversal of the net protein charge (new Fig. 3).

These results indicate that the spectral shift cannot be explained by refractive-index loading or simple net electrostatic charge effects.

Reviewer 3 Comment 3

"The reviewer suggests performing simpler charge-controlled experiments or demonstrating switching of the blue shift."

Response:
We have introduced additional experimental controls to probe the role of charge and structure:

- **pH variation (new Fig. 3):** The spectral shift persists across the isoelectric point.
- **Native vs. denatured comparison (new Fig. 5):** While spectral shifts are observed in both cases, the handedness-dependent linear dichroism (LD) differs between native and denatured states.

These experiments indicate that the optical response is not solely governed by surface charge accumulation or nonspecific adsorption.

Reviewer 3 Comment 4

"The reviewer notes that Figure S2 and related panels are difficult to read and suggests improving clarity."

Response:
All supplementary figures have been reformatted at higher resolution. Spectra have been reorganised for improved clarity, and figure references in the main text have been corrected.

Response to reviewer's comments

(Questions/comments are in **bold**)

Reviewer #1 (Remarks to the Author):

The authors have made substantial structural and conceptual revisions to the manuscript. The introduction of a rigorous electromagnetic boundary-condition formulation successfully provides a quantitative basis for interpreting the observed spectral behavior. The authors have thoroughly addressed my previous concerns and incorporated valuable new experimental data, including conformational controls and measurements across the isoelectric point. These additions significantly elevate the scientific rigor and logical consistency of the work. However, a minor inconsistency persists in Figure 4. In the caption for Figure 4, the text states, "The R parameter is defined in the supplementary information...". The accompanying table in Figure 4d defines the asymmetry parameter using the symbol α (e.g.,), and the main text refers to "peak R" as the resonance associated with the magnetic-dipole mode. The authors should correct the caption to refer to the "α parameter" or "asymmetry parameter" rather than the "R parameter" to avoid confusing the parameter itself with the label for the resonance peak. Overall, I would like to recommend acceptance of the manuscript after the minor typographical issue noted above is addressed.

Author response: We thank the reviewer for appreciating the improvement. We have modified the manuscript as suggested by reviewer and fix the typo in Figure 4. We have replaced the R parameter with "α" parameter and defined it in supplementary information.

Reviewer #2 (Remarks to the Author):

The revised manuscript demonstrates a substantial improvement in both scholarly completeness and overall contribution relative to the original submission. As detailed in the authors' Response to Referees, the manuscript has been extensively revised at both the structural and conceptual levels. Notably, the concept of the "meta-diastereomer," which was a shared concern among the reviewers, is now presented with much greater clarity. The experimental design and interpretation of the results have also been significantly strengthened, and the newly added computational simulations are in much closer agreement with the experimental findings. Consequently, the main contribution of the work has been successfully repositioned from the originally proposed biosensing platform toward the identification of a boundary-mediated mechanism through which

molecular chirality is translated into the optical response. In this respect, the manuscript is now much improved and, in my view, is potentially suitable for publication after minor revision. The remaining issues are primarily related to clarity of presentation and consistency of notation.

Author response: We thank the reviewer for appreciating the work. We have considered the comments for improving the quality of the manuscript, and they are addressed below.

1. On Page 5, Line 2, the authors state that even when the same fabrication process is employed, the silicon thickness varies slightly within the range of 170–210 nm. This variation is entirely understandable, and the authors have appropriately specified the silicon thickness used in each experiment when describing the results in Fig. 3, Fig. 5, and related sections, which improves the rigor of the manuscript. However, in the current presentation, readers may still be left uncertain as to whether the differences in silicon thickness reported across experiments simply reflect unavoidable sample-to-sample variation arising from the fabrication process, or whether silicon thickness was intentionally controlled as an experimental variable. It would therefore be helpful if the descriptions of the silicon thickness used in the experiments were revised throughout the manuscript in a more explicit and reader-friendly manner.

Author response: We thank the reviewer for identifying the lack of clarity in explaining the thickness variation of the samples. We have modified the above-mentioned part of the manuscript for the clarity and mentioned that it's result of uncontrolled variation across different run. It's modified to "While we optimise the fabrication process for a thickness of 180 nm, actual thickness ranges from the 170-210 nm across different runs. Although these unavoidable sample-to-sample variations produce spectral shifts and line-shape differences, they do not affect the qualitative behaviour described below."

2. On Page 5, Line 11, the manuscript states: "Absorbance (Abs) and depolarization Index (0≥DI≤1) ...". This appears to be a typographical error and presumably should read "(0≤ DI≤1)". Likewise, on Page 5, Line 13, "0.5≥DI≤1" also appears to be a typographical error and likely should read "0.5≤DI≤1". If so, these expressions should be corrected.

Author response: We have fixed this typo in the manuscript and manuscript has been revised thoroughly.

3. In Fig. 3, the inclusion of an appropriate legend would improve readability. At present, the measurement conditions corresponding to each graph are described only in the figure caption, which makes the figure somewhat less intuitive to interpret. In addition, all of the

graphs are presented in the same color (red), which further reduces visual clarity. I therefore suggest using clearly distinguishable colors for the different graphs and including a corresponding legend directly within the figure.

Author response: We have added the legend in figure 3 for improved readability and clarity.

4. In Fig. 4a–c, the x-axis ticks should be indicated more clearly. Currently, only Fig. 4c provides numerical labels for the x-axis range and tick positions, and even there, some graphs appear to show slight inconsistencies between the grid and tick positions. I therefore recommend that the x-axis ticks in each of Fig. 4a–c be presented more clearly and accurately.

Author response: We have edited figure 4a and labelled the X-axis properly. We have also removed the gridlines from the graph for improved readability.

5. On Page 10, Line 4, "fig. 4d" is written in lowercase. For consistency with the notation used throughout the manuscript, I suggest revising this to "Fig. 4d".

Author response: We have edited the manuscript thoroughly and fixed the typos suggested by reviewer and editorial team. We have thoroughly revised the Figure 4d.

Reviewer #3 (Remarks to the Author):

I read the response of the authors to the reviewers' comments and the revised manuscript and I find the paper significantly improved in the clarity of the proposed model and physical decription of it.

I support publication, since I do not have a better explanation for the blue shift, although I am still not totally convinced about the boudary layer model. I would have still loved to see some more experimental proof for it. For example, considering eq. 3, I would assume that if the protein at the interface would have been replaced with a metal nanoparticle film, wouldn't the high polarizability of the metal increase the polarization effect significantly? in that case wouldn't the blue shift become more substantial? Or maybe it will scramble the whole reflectance spectrum completely, and then it will not be so useful? Just a thought to consider in order to strengthen the proposed model.

Another point: A possible explanation about the difference in optical responses between the chiral liquid and the proteins could be due to aligned adsorption vs. total randomness in chiral butanol molecules orientation. I know that the authors claim that the

glutaraldehyde process does not leave the protein aligned with respect to the surface, but I am not sure if this is correct.

Figure 3b: the dotted line for the adsorbed strep cannot be seen in the graph.

Author response: We thank the reviewer for pointing this out. We have modified the figure 3, changed the colour of the graph, added the legend and removed the gridlines for the better visibility.
